# A New Perspective in Epileptic Seizure Classification: Applying the Taxonomy of Seizure Dynamotypes to Noninvasive EEG and Examining Dynamical Changes across Sleep Stages

**DOI:** 10.1523/ENEURO.0157-24.2024

**Published:** 2025-01-08

**Authors:** Miriam Guendelman, Rotem Vekslar, Oren Shriki

**Affiliations:** Departments of ^1^Cognitive and Brain Sciences; ^2^Computer Science, Ben-Gurion University of the Negev, Beer-Sheva 8410501, Israel

**Keywords:** automated seizure classification using machine learning, bifurcations in epilepsy, epileptic seizure classification, noninvasive EEG analysis, sleep stages and seizure dynamics, taxonomy of seizure dynamotypes (TSD)

## Abstract

Epilepsy, a neurological disorder characterized by recurrent unprovoked seizures, significantly impacts patient quality of life. Current classification methods focus primarily on clinical observations and electroencephalography (EEG) analysis, often overlooking the underlying dynamics driving seizures. This study uses surface EEG data to identify seizure transitions using a dynamical systems–based framework—the taxonomy of seizure dynamotypes—previously examined only in invasive data. We applied principal component and independent component (IC) analysis to surface EEG recordings from 1,177 seizures in 158 patients with focal epilepsy, decomposing the signals into ICs. The ICs were visually labeled for clear seizure transitions and bifurcation morphologies (BifMs), which were then examined using Bayesian multilevel modeling in the context of clinical factors. Our analysis reveals that certain onset bifurcations (saddle node on invariant circle and supercritical Hopf) are more prevalent during wakefulness compared with their reduced rate during nonrapid eye movement (NREM) sleep, particularly NREM3. We discuss the possible implications of our results in the context of modeling approaches and suggest additional avenues to continue this exploration. Furthermore, we demonstrate the feasibility of automating this classification process using machine learning, achieving high performance in identifying seizure-related ICs and classifying interspike interval changes. Our findings suggest that the noise in surface EEG may obscure certain BifMs, and we suggest technical improvements that could enhance detection accuracy. Expanding the dataset and incorporating long-term biological rhythms, such as circadian and multiday cycles, may provide a more comprehensive understanding of seizure dynamics and improve clinical decision-making.

## Significance Statement

Traditional seizure classification focuses on clinical symptoms and electrophysiological signs but often overlooks the underlying seizure dynamics. The taxonomy of seizure dynamotypes introduces a novel computational approach that links electrophysiological transition signatures to these dynamics. While previously applied to invasive recordings, this study extends the taxonomy to noninvasive electroencephalography. Our analysis reveals a relationship between sleep stages and seizure dynamics. We suggest that integrating these modeling approaches with sleep and circadian dynamics models may reveal insights into seizure timing and generalization, opening new pathways for better diagnostics. Broader adoption of this classification is limited by its labor-intensive visual inspection process. Here, we demonstrate the potential of automated classification, enabling analysis to scale to larger cohorts.

## Introduction

Epilepsy, characterized by recurrent unprovoked seizures, affects millions worldwide and presents a significant challenge in neurology. Seizure classification approaches rely primarily on clinical observations and electroencephalography (EEG) visual analysis. Yet, in most cases, this classification does not provide information on the underlying dynamics. A better understanding of the transitions into and out of a seizure can shed light on the mechanisms that drive seizure activity.

From a dynamical systems point of view, the brain is constantly shifting between dynamical states in response to internal fluctuations and external stimuli (Deco et al., 2011). Epilepsy is viewed as a condition that facilitates transient oscillatory ictal states (Da Lopes Silva et al., 2003), and the transition into and out of a seizure can be considered a bifurcation—a mathematical concept that describes a sudden change in the system's dynamics. This offers a new lens through which to analyze the initiation and termination of seizures, potentially leading to novel strategies for their treatment.

Through rigorous mathematical and modeling work, research in this area has mapped out the “taxonomy of seizure dynamotypes” (TSD), introducing a structured classification approach that is based on a minimal descriptive model, connecting electrophysiological morphologies to dynamical bifurcations (Saggio et al., 2020). In this paper, we will refer to these as bifurcation morphologies (BifMs). Similar models have demonstrated clinical relevance by improving the outcome of resective surgery (An et al., 2019; Makhalova et al., 2022) or predicting the effect of electrical stimulation on seizure abortion (Szuromi et al., 2023).

The TSD approach describes seizure transitions using several time scales: a fast subsystem that intermittently generates seizure activity; a slow subsystem that modulates the fast system's parameters, facilitating mainly seizure termination; and an ultraslow subsystem that can effect seizure propensity (Saggio et al., 2020). From this modeling perspective, the fast subsystem represents the seizure onset zone (SOZ); the slow subsystem represents reactive processes limiting seizure duration; and the ultraslow subsystem corresponds to changes in the brain state that occur over significantly longer timescales. The last, which is not explicitly modeled in the TSD framework, may include sleep stages (Herman et al., 2001), as well as circadian or longer physiological rhythms, which have been shown to affect seizure propensity (Karoly et al., 2021). Sleep stages, particularly wake, nonrapid eye movement (NREM), and REM, represent inherently different dynamical states with different tendencies toward epileptic events (Ng and Pavlova, 2013; Halász et al., 2019), making them a significant target for investigation.

The TSD framework has been investigated using invasive data from animal models and human subjects (Jirsa et al., 2014; Saggio et al., 2017, 2020). Previous research explored the relationship between factors such as age, gender, etiology, and localization (Saggio et al., 2020); however, this analysis was limited to a single seizure per patient and did not account for sleep stages. Analyzing multiple seizures per patient presents several statistical challenges, including accounting for repeated measures within patients and considering both patient- and seizure-level factors. Additionally, the inherent imbalance in BifMs introduces further statistical complexities.

As noted above, previous research has primarily relied on invasive intracranial recordings, which, while effective, are limited in broader application due to their invasiveness. In contrast, surface EEG is more commonly used in epilepsy diagnosis, but it is often compromised by significant noise from nonbrain sources, particularly during seizures. To mitigate this issue, we have employed signal decomposition methods such as principal component analysis (PCA; Maćkiewicz and Ratajczak, 1993) and blind source separation techniques like independent component analysis (ICA; Bell and Sejnowski, 1995) to extract seizure-related information from the EEG signal. These methods have proven effective in tasks like focal seizure localization (De Vos et al., 2007; Stern et al., 2009; Matin et al., 2019; Rabby et al., 2021). Notably, a strong temporal and spatial correlation between noninvasive independent components (ICs) and intracranial recordings from the SOZ has been reported (Barborica et al., 2021). Therefore, selecting ICs that contain seizure information may provide a reasonable approximation of the activity generated in the SOZ.

In this study, we explore a possible methodology for extracting seizure information from surface EEG by performing a visual analysis of the resulting ICs, identifying ICs of brain origin with clear seizure transitions, and classifying the BifMs. We then examine the relationship between BifMs and clinical factors at the patient and seizure levels using Bayesian multilevel modeling (Bürkner, 2017). Recognizing that the labor-intensive process of visually classifying BifMs limits scalability, we aimed to automate the identification of seizure-related ICs and the classification of different BifMs to enhance the method's applicability.

## Materials and Methods

### Data and selection criteria

**Figure 1.**
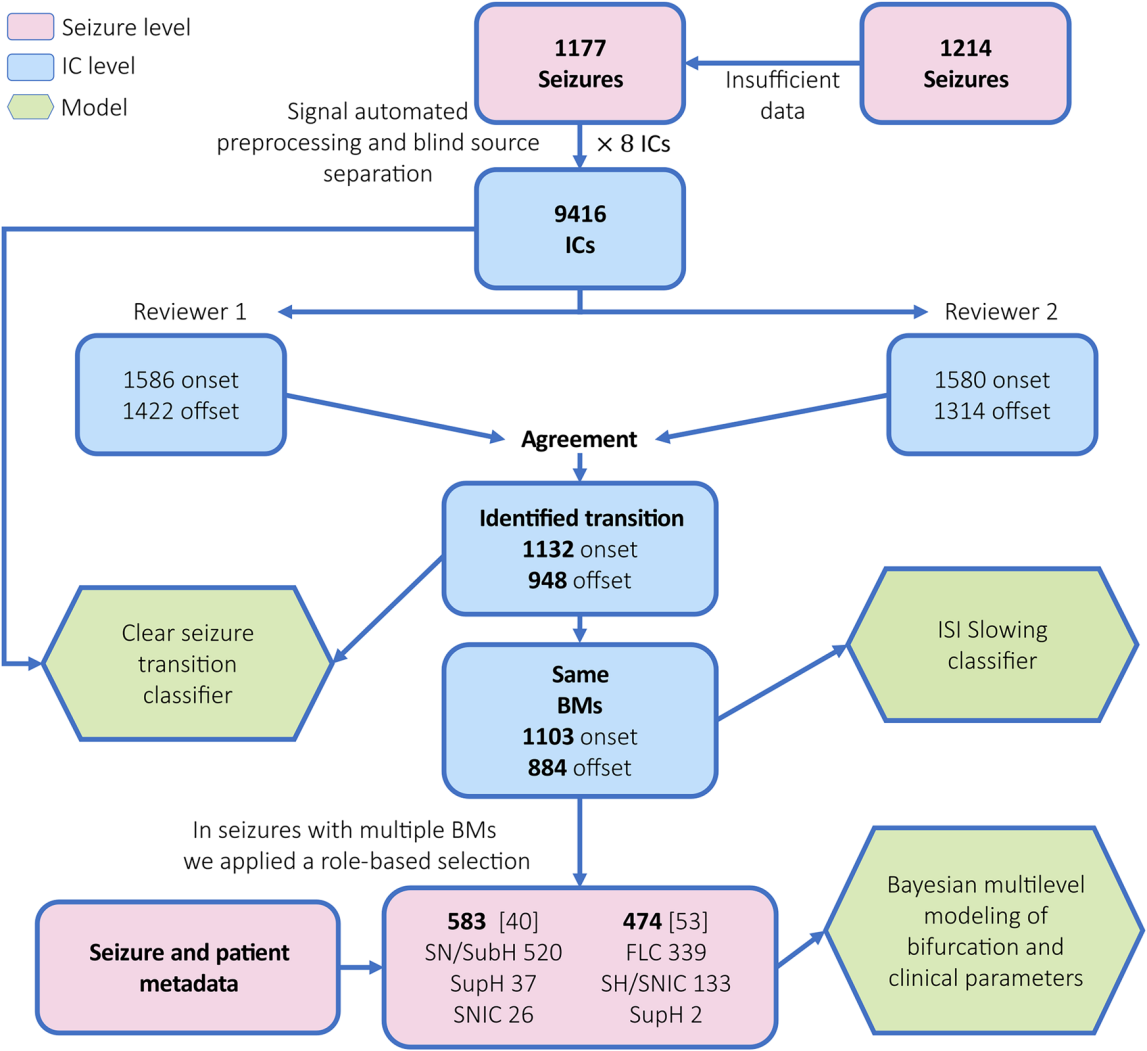
Analysis overview. Project overview: we utilized data from the EPILEPSIAE surface database (Ihle et al., 2012), which includes 1,214 seizures from 158 patients undergoing presurgical evaluation. Using the seizure annotations provided in this database, we automatically extracted and preprocessed the seizure data, adding a margin of 30% of the seizure length on each side. Thirty-seven seizures lacking sufficient data were excluded during preprocessing. The preprocessing pipeline is detailed in Materials and Methods, Visual analysis and labeling. Initially, we reduced the dimensionality of the EEG data using PCA to retain the top eight components. ICA was then applied to achieve signal components with strong statistical independence, resulting in 9,416 components. Two independent raters visually reviewed these components, with inter-rater agreement evaluated (Tables 1, 2). Only components with agreement were used for further analysis. During the visual analysis, we focused on identifying components with clear transitions into and out of a seizure agreed upon by both reviewers and classifying the transition's BifM based on the “TSD” (Saggio et al., 2020). These agreed-upon components served as the gold standard for developing two automated classifiers. We evaluated the models using a LOOCV approach (detailed in Materials and Methods, Development and validation of automated classification; Fig. 4; Tables 5, 6; Extended Data Tables 5-1, 6-1). A seizure could contain multiple components with clear transitions (Fig. 2*A*). We used objective measures extracted from all components to select the one that best represented the onset and offset for each seizure, primarily based on the transition timing and the probability of containing brain information (see Extended Data Fig. 2-2 for more details). Most seizures included a single bifurcation, but we recorded cases where more than one bifurcation type was initially labeled (detailed in Table 1). We modeled the prevalence of seizure BifMs in relation to various seizure factors (vigilance, seizure length, and classification) and patient factors (anatomical localization, age, gender, etiology) using a Bayesian multilevel model (for more details, see Materials and Methods, Modeling the interactions between BifMs and clinical factors). The overall BifM prevalence (Fig. 3) served as the prior probability for the model intercept initialization. We used the posterior estimations to examine the differences between modeled factors. Extended Data Figure 4-1 explains the metrics used to compare the posterior distributions. The analysis results are detailed in Table 4 and in Extended Data Tables 4-1 and 4-2.

We utilized the EPILEPSIAE database (Ihle et al., 2012), which provides meticulously labeled seizure onset and offset times for EEG recordings from 158 patients with drug-resistant focal epilepsy undergoing presurgical evaluation. This database includes 1,214 seizure annotations (Fig. 1, top right). For each seizure, a control segment of identical duration was selected, positioned at least 5 min away from any seizure activity within the same recording. Due to insufficient control data, 38 seizures were excluded, resulting in a total of 1,177 seizures that were visually analyzed (Fig. 1, top middle).

10.1523/ENEURO.0157-24.2024.t1-1 Table 1-1 The table includes all the patient and seizure metadata for the 1,177 seizures included in the analysis. Download Table 1-1, XLSX file.

10.1523/ENEURO.0157-24.2024.t1-2 Table 1-2 The table includes the reviewer labels for all ICs and the final labels determined by inter-rater agreement. Download Table 1-2, XLSX file.

Seizure annotations included the sleep stage before a seizure and seizure classification. In this database, seizures were classified, according to the 1981 classification scheme (Bancaud et al., 1981), as simple partial (focal aware, FAS), complex partial (focal with impaired awareness, FIAS), and secondarily generalized (focal to bilateral tonic–clonic, FBTCS). Unclassified (UC) seizures remained the same (Scheffer et al., 2017). Patient metadata included age, gender, seizure localization, and etiology. Patient ages ranged from 13 to 65 years, with a mean of 35.4 years; there were 82 male and 76 female patients. The most common seizure localization was in the temporal lobe (117), followed by the frontal lobe (17). The most common etiology was hippocampal sclerosis (54), followed by cryptogenic (46) and cortical dysplasia (28). Extended Data Table 1-1 provides detailed patient and seizure metadata used in this work.

### Preprocessing for the extraction of seizure information from surface EEG data

Preprocessing was conducted using MATLAB and EEGLAB (Delorme and Makeig, 2004). The code in this work was run on a 64 bit operating system, an x64-based processor, and a Windows 10 pro operating system to obtain the results reported here. The EEG signals were downsampled to 256 Hz to ensure uniformity across the database. A high-pass filter with a 1 Hz cutoff was applied to remove low-frequency noise and drift. We utilized artifact subspace reconstruction (Mullen et al., 2015) to identify and correct strong movement artifacts in the recordings. After artifact correction, the signals were re-referenced to the mean, and a low-pass filter with a 40 Hz cutoff was applied.

Under the assumption that synchronous ictal EEG activity accounts for a significant portion of signal variance, we applied PCA (Maćkiewicz and Ratajczak, 1993). PCA transforms the data into a new set of variables called principal components (PCs), which are orthogonal (uncorrelated) and ordered by the amount of variance they capture. By selecting the top PCs, we reduced the dimensionality of the data while preserving most of the variance.

EEG data often exhibit significant amplitude variations due to differences in electrode impedance, contact, and positioning, which can bias the PCA results. For example, the first PC may be mainly influenced by the electrode with the largest amplitude. This could be due to either synchronized ictal activity or technical reasons (e.g., movement of an electrode). To address this issue, we standardized the electrode data with a 30% margin on each side of the seizure annotation. Including these edges in the standardization process (mean reduction and unit scaling, also termed pre-whitening) preserves the relative amplitude increase associated with ictal activity, which might otherwise be diminished if only ictal data were used.

In the next step, we applied infomax ICA on the resulting PCs (Bell and Sejnowski, 1995). ICA is used to find a linear transformation that makes the output signals as statistically independent from each other as possible. It is commonly used in EEG analysis to separate and remove noise sources such as eye movements, muscle activity, and heartbeats (Delorme and Makeig, 2004). Previous research has demonstrated that ICA can be effectively applied to surface EEG data to extract ICs that exhibit strong temporal and spatial correlations with ictal intracranial recordings from the SOZ (Barborica et al., 2021).

**Figure 2.**
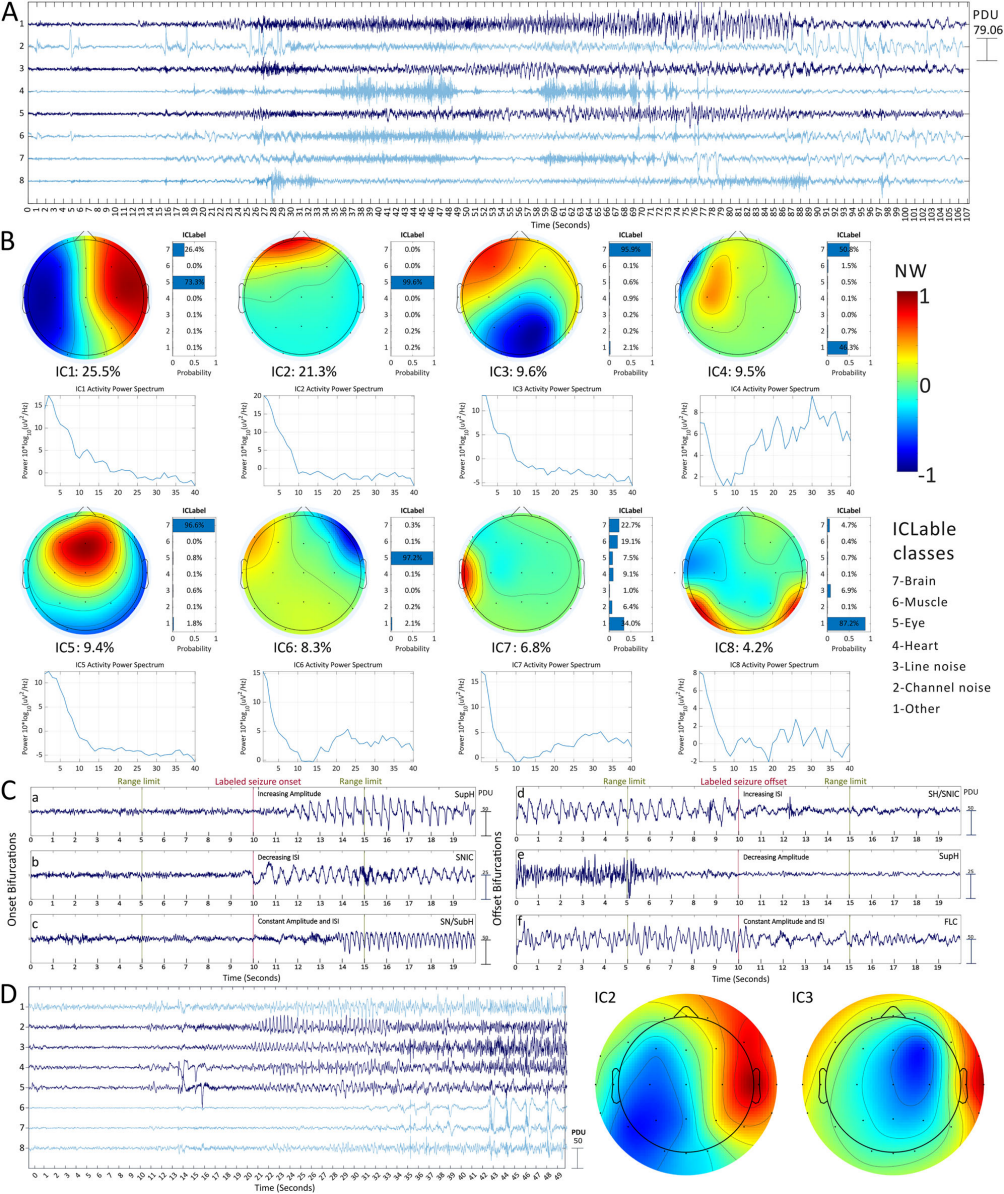
Information used to label the ICs. ***A***, The time series of the ICs from an example seizure. The ICs highlighted in dark blue contain a clear transition into a seizure that includes data mainly from brain sources. The PCA–ICA procedure affects the components’ scale; hence, the units are presented as procedure-defined units (PDUs). ***B***, For the selection of the ICs, we used custom code and EEGLAB visualization functions to present each reviewer with the component time series, the topographic distribution of the electrode weights, the spectral profile, and the IClabel classifier output. The topographic maps represent the normalized weight (NW) distribution used to transform the EEG time series into the IC time series. These maps help evaluate the scalp distribution of each component, helping with the recognition of patterns typical for brain-related sources (IC1, 3, 5), eye movement (IC2, 6), or other nonbrain sources (IC4, 7, 8; as described in Pion-Tonachini et al., 2019b). Using the power spectrum, we could better distinguish ICs with typical brain frequency profiles (IC1, 3, 5) from components containing mainly muscular activity (IC4, 6, 8). These and additional signal features are input to the IClabel classifier. This classifier is built into EEGLAB and is designed to identify and remove noise components in surface EEG preprocessing (Pion-Tonachini et al., 2019a). To support our decision and to estimate the consistency of this classifier on seizure data, we presented the classification probabilities to the user. Extended Data Figure 2-2*A* displays the overall brain score distribution given the different labels and shows that clear transitions are generally associated with a higher brain score. However, exceptions exist, and this does not provide a clear-cut role to identify seizure data. ***C***, Examples of the three onset BifMs: (***a***) SupH, which shows an amplitude increase (from zero); (***b***) SNIC, characterized by increased spike frequency (from zero) or decreasing ISIs from the seizure onset; and (***c***) SN (expected DC shift) or SubH, in which both amplitude and ISI remain constant or random; however, due to the lack of DC recording, these are indistinguishable. Examples of the three offset BifMs include (***d***) SNIC or SH (expected DC shift), characterized by increasing ISIs or reduction in frequency (to zero), and these are indistinguishable like the previous example; (***e***) SupH, with an amplitude decrease (to zero); and (***f***) FLC, with amplitude and ISI that are constant or random toward seizure offset. See Extended Data Figure 2-1 for additional examples. ***D***, An increasing amplitude at the seizure onset characterizing the SupH BifM may be attributed to propagation in noninvasive EEG. Therefore, it is important to analyze the component time series in the context of additional information beyond the time series alone. For example, if we observe only IC2, it shows a SupH BifM starting at the 20 s mark. However, when examining IC3, we see a lower constant amplitude beginning at the 16 s mark and undergoing a second change at the 20 s mark. The topographic maps of these components reveal a more localized weight distribution in IC3 than in IC2. This suggests that the pattern in IC2 may result from propagation, emphasizing the importance of selecting the earliest and most localized spatial pattern (in focal seizures) as the seizure onset BifM.

The PCA and ICA steps produce two linear transformation matrices that convert the prewhitened EEG electrode data into ICs. By multiplying these matrices, we can present the electrode weights for each component, which helps identify the relative contribution of surface electrodes to each IC, as shown in Figure 2*B*. A preliminary analysis of 10 randomly selected seizures indicated that seizure information was concentrated within the first eight PCs. Using more than eight PCs introduced noise and resulted in less distinct ICs with clear seizure information. Therefore, we established a cutoff of eight PCs to minimize noise and reduce the workload for visual labeling, leading to a total of 9,416 components (Fig. 1, top middle).

10.1523/ENEURO.0157-24.2024.f2-1 Figure 2-1 Labeling examples. Each component is a linear combination of all electrodes with varying weight ranges; hence, we present the component time series with procedure-defined units (PDUs). Download Figure 2-1, TIF file.

10.1523/ENEURO.0157-24.2024.f2-2 Figure 2-2 Automated measures and detectability characteristics. Download Figure 2-2, TIF file.

### Visual analysis and labeling

Before initiating the visual analysis and labeling, two independent reviewers were trained on the classification tasks. The initial challenge was identifying ICs containing brain activity related to seizures in surface EEG data. To improve proficiency in distinguishing between brain and nonbrain sources, reviewers used the ICLable labeling tutorial application (Pion-Tonachini et al., 2019b). This step was crucial for enhancing the ability to identify various physiological and nonphysiological IC sources. Figure 2, *A* and *B*, illustrates the primary information sources used for labeling.

Reviewers were also trained to identify seizure onset and offset BifMs using empirical and simulation examples from previous work (Saggio et al., 2020). This classification was based on specific trends in frequency (interspike intervals, ISIs) and amplitude changes. In general, there are six types of BifMs: supercritical Hopf (SupH), saddle node on invariant circle (SNIC), saddle homoclinic (SH), saddle node (SN), subcritical Hopf (SubH), and fold limit cycle (FLC).

The SupH BifM prescribes an amplitude scaling law where the amplitude increases from zero at the onset and decreases to zero at the offset. SNIC and SH BifMs are characterized by a spike frequency scaling law. In the SNIC BifM at the onset, the frequency increases from zero. At the offset, SNIC and SH BifMs are characterized by frequency decreasing to zero. Finally, SN and SubH BifMs at the onset and the FLC BifM at the offset have no prescribed scaling laws and may exhibit constant or arbitrary amplitude and spike frequency. Detailed examples and expected features of each BifM are provided in Figure 2*C*.

The absence of a direct current (DC) component in our data limited the distinction between SN and SubH BifMs at the onset, as well as between SH and SNIC BifMs at the offset. As a result, we grouped these BifMs into combined categories, following the approach used in previous studies (Saggio et al., 2020). The onset labels were categorized as SupH, SNIC, SN/SubH, and unclear, and the offset labels were defined as SupH, SNIC/SH, FLC, and unclear.

We developed an in-house graphical user interface in which reviewers were presented with the EEG time series, the eight ICs’ time series for each seizure, their spatial activation maps, and the power spectrum (see Fig. 2*A*,*B* for additional details). This interface provided a context for selecting the most relevant ICs, which contained both brain activity and clear transitions into and out of seizures; these were then labeled with the relevant BifM. Each reviewer labeled the ICs from the 1,177 seizures—a total of 9,416 ICs (Fig. 1, IC level). Figure 2*C* provides labeling examples and considerations, with additional examples provided in Extended Data Figure 2-1.

After the first labeling session, the reviewers recognized a learning curve in distinguishing brain-derived data from other physiological sources within seizure data. Components not unanimously agreed upon as seizure-related were independently relabeled by each reviewer, who was blinded to the prior labels. Components that were unanimously identified as either seizure-related or not, despite differences in BifM labels, were not relabeled. The top three rows in Table 1 summarize the ICs labeled by both reviewers and the number of components with agreements at the onset and offset.

**Table 1.** Component agreement numbers: this table includes details on the numbers of ICs and seizures included throughout the analysis

	Reviewer 1	Reviewer 2	Clear transition	BifM with agreement	Multiple BifMs (single seizure)
ICs reviewed overall	9,416	9,416			
Offset ICs	1,422	1,314	948	884	
Onset ICs	1,586	1,580	1,132	1,103	
Seizures reviewed overall	1,177	1,177			
Seizures with clear offset	568	565	505	474	53
Seizures with clear onset	634	651	598	583	40
Offset ICs per seizure	2.503521	2.325664	1.877228	1.864979	
Onset ICs per seizure	2.501577	2.427035	1.892977	1.891938	

BifM, bifurcation morphology. All labels are provided in Extended Data Table 1-2.

We quantified the inter-rater agreement using Cohen's kappa coefficient (Cohen, 1960), evaluating it separately for ICs identified as having a clear transition and for the BifMs labeled. To estimate the chance level for kappa values given the label imbalance, we conducted two permutation analyses. The first approach involved randomly permuting the reviewers’ labels overall, while the second involved permuting labels only within each seizure, ensuring the same number of ICs labeled by each reviewer per seizure. Each analysis included 10,000 permutations. Following this agreement evaluation, only ICs marked with a similar bifurcation by both reviewers were considered to be clear bifurcations and were included in further analysis.

In some instances, more than one IC per seizure was labeled as having a BifM. Typically, the ICs for each seizure shared the same BifM; however, there were several seizures (40 onsets and 53 offsets) where multiple ICs were labeled with different BifMs. In these cases, we determined the BifM at the seizure level by evaluating objective measures such as the transition time and a general brain score. For a more detailed description of these measures and the decision flow, see Extended Data Figure 2-2*A–C*.

### Modeling the interactions between BifMs and clinical factors

To analyze the relationship between BifMs and clinical factors at the patient and the seizure levels, we used the BRMS package (Bürkner, 2017) in R, which implements Bayesian multilevel models. These statistical models allow us to account for data hierarchies and repeated measures within a patient. Moreover, Bayesian models provide a probabilistic framework for statistical inference, incorporating prior knowledge and the observed data to estimate the parameters of interest.

Patient-level effects included age, gender, the lobe of the epileptogenic zone, and etiology. We treated the patient as a random effect for vigilance, seizure length, and classification to account for repeated measures within subjects. We included all three BifMs—SN/SubH, SNIC, and SupH—in a categorical logistic regression model for the onset. However, we encountered only three seizures with a SupH BifM for the offset. Consequently, we created a binomial model that included only SH/SNIC and FLC BifMs, excluding seizures with SupH BifM from the analysis.

In Bayesian statistics, a prior represents our initial belief about the parameters. Here, the intercept priors reflect our expectations about the BifM distribution. By initializing the model intercepts with the overall bifurcation distribution, we effectively assume a particular and similar BifM distribution across the clinical factors. Consequently, more robust evidence is required to deviate from this initial distribution during the model fitting process. This approach helps prevent overestimating effects due to random variations and makes our conclusions more robust.

The model distribution parameters were estimated using Monte Carlo–Markov chain (MCMC). MCMC is a computational algorithm used to approximate the posterior distribution when it is difficult to calculate directly. After considering the observed data, the posterior distribution represents our updated beliefs about the parameters. For each model, we used six chains with 6,000 iterations each; the first 2,000 iterations were considered a warm-up (or burn-in period) and were excluded from the final posterior estimation to ensure that the chains had reached a stable distribution.

For adequate model fitting, we evaluated the fit quality in two ways. First, we performed a posterior predictive check (PP check) by simulating the overall posterior distribution and confirming that it accurately represented the observed data. This involves generating and comparing data from the model to the actual data to assess how well the model captures the underlying patterns. Second, we assessed the $\hat{R}$ statistic, which measures the convergence between and within chains; an $\hat{R}$ value >1 indicates poor mixing and unreliable posterior estimates (Vehtari et al., 2021); in our models, $\hat{R}$ was equal to 1.

The Bayesian modeling approach allowed us to estimate posterior distributions under two clinical conditions, marginalized across all other factors, and to analyze the difference distribution to estimate the direction and effect size. For a centrality measure, we report the median and maximum a posteriori (MAP) of the difference distribution function and the credible interval (CI), which is equivalent to the frequentist confidence interval and provides a range within which the parameter value lies with a 0.95 probability (Extended Data Fig. 4-1*A*). To estimate the magnitude of the effect, we use the probability of direction (pd) (Extended Data Fig. 4-1*B*). The pd is mathematically defined as the proportion of the posterior distribution on the side of the median, effectively quantifying the certainty about the direction of an effect. It ranges between 0.5 (maximum uncertainty) and 1 (maximum certainty). A pd larger than 0.97 is considered to be a strong effect (Makowski et al., 2019).

We also used the full region of practical equivalence (ROPE) to define the null hypothesis over a range of values considered negligible or too small to be practically important. The ROPE approach allows us to determine whether an effect is not only strong but also if it is practically meaningful. This measure quantifies the percentage of the posterior distribution within the ROPE range (Extended Data Fig, 4-1*C*). In this study, we defined a 10% change in the minority class as the range of practical equivalence. A ROPE >0.97 indicates strong support for the effect being practically negligible, while a ROPE <0.03 indicates a meaningful effect size (Makowski et al., 2019).

### Development and validation of automated classification

#### Feature extraction

All features were extracted utilizing a custom MATLAB code. The offset signal was flipped on the time axis to align the nonictal to ictal segments and trends with the onset data. We applied the IClabel (Pion-Tonachini et al., 2019a) and MARA (Winkler et al., 2011) classifiers on the ICs, both globally for each IC and locally around the onset and offset bifurcations. Extended Data Figure 2-2*A* descriptively illustrates that components labeled with a clear BifM had a higher mean brain probability estimate from these classifiers, indicating the potential utility of this information in detecting ICs containing seizure-related data.

For precise detection of the transition time, we analyzed a 10 s window around the seizure annotations (both onset and offset) and the corresponding time periods in the control datasets. Our goal was to identify an objective shift in the statistical characteristics of the signal, referred to as a change-point (Truong et al., 2020). We used a variance-based change–point detection algorithm (MATLAB's “ischange” function) and restricted the detection to a single change-point. When a single clear change-point was identified, it served as an anchor for subsequent feature extraction, significantly improving the quality of the features, especially for estimating amplitude and ISI changes. If no change-point was found, the dataset annotation time was used as the reference start point.

A change-point can sometimes occur outside a seizure or be related to nonbrain changes within a seizure; however, Extended Data Figure 2-2*B* shows a higher proportion of detected change-points in seizure data, particularly in ICs selected for having clear BifMs. A limitation of this approach is that if the change occurs close to the edge of the analysis window (less than a second), it may not be detected. Extending the analysis time to accommodate this could introduce nonstationarity biases, so we opted not to extend the analysis window.

Amplitude modulation was assessed using the peak amplitude and the signal's envelope root mean square during the first 2 s of the transition time. ISIs were determined as intervals between subsequently identified peaks within 5 s of the estimated transition time during the seizure. Established morphological curves (Saggio et al., 2020) were fitted to these values, with the fit quality assessed via root mean square error (RMSE).

Over a 5 s window from the transition time within the seizure, we computed the signal's mean, median, standard deviation, and skewness. For ISIs, both the mean interval and standard deviation were calculated. The power spectrum of the signal, ranging from 1 to 40 Hz, was computed. A linear fit was applied to the logarithm of the power spectrum against frequency, from which intercept, slope, confidence intervals, RMSE, and adjusted *R* squared were derived. The most deviant positive point from the linear fit line was set as the frequency peak, potentially correlating with the dominant seizure oscillation frequency.

Within the same time window, the spectral and approximate entropy—recognized as robust seizure detection metrics (Boonyakitanont et al., 2020)—were employed to pinpoint seizure-associated ICs. The signal's autocorrelation function was used to delineate cyclical patterns. The peaks in the autocorrelation function were identified, and the intervals between them were analyzed to compute their mean and standard deviation. The entropy of the autocorrelation function was calculated to provide insights into signal oscillation regularity. Additionally, the width of the autocorrelation function, a metric associated with critical slowing down (Maturana et al., 2020), was estimated as it could be related to the proximity to a bifurcation event at larger time scales.

An initial evaluation of the extracted features was conducted to address various considerations. In several instances, feature extraction failed due to brief seizure durations or inadequately identified peaks, often attributed to noise interference that resulted in missing values. For each classification task, we excluded features with over 5% missing values.

#### Automated classification

We separated our classification task into two stages. Initially, we focused on finding ICs that included a clear transition into or out of a seizure. Subsequently, we aimed to classify the BifM features within these ICs. The two key morphological features are the amplitude and ISI changes. In surface EEG, the amplitude may not solely reflect seizure-initiating neuronal properties but could also indicate the extent of the recruited neuronal population as the seizure propagates (Fig. 2*D*). Moreover, focusing on a binary classification task facilitates a more straightforward model evaluation and interpretation. Therefore, we first prioritized automating the classification of ISI change.

Developing a proper classifier for these tasks involved three main challenges. The first was the limited sample size, which heightened the risk of overfitting and reduced the potential for generalization. To mitigate this, we combined onset and offset samples to increase the sample size and integrated a categorical feature to account for inherent differences between the two groups. We used a random forest (RF) model, limiting the maximum tree depth to three to reduce overfitting (Fawagreh et al., 2014). In the context of the RF model, the categorical feature allows the model to learn distinct decision flows for onset and offset transitions, preserving the distinction between these two groups in the classification task. At the same time, this approach enables the classifier to leverage common patterns shared between onset and offset cases. The second challenge was the noisy nature of the seizure data, which included potential outliers. To address this, we employed the RobustScaler from scikit-learn (Pedregosa et al., 2011), providing a scaling approach that is robust to outliers. In regard to the final challenge, the class imbalance in our data, which could bias the classifier toward the majority class, we recalibrated the class weights to ensure equal sensitivity across both classes (Sun et al., 2009).

We evaluated performance using a leave-one-subject-out cross–validation (LOOCV) paradigm, which allowed us to assess performance across patients and estimate performance confidence intervals (Tougui et al., 2021). We measured performance using balanced accuracy (BA; Brodersen et al., 2010) across all patients. The area under the receiver operating characteristic curve (AUC-ROC) was computed for patients with both classes present. However, this computation was not feasible for patients with only one class, so it was not reported in these cases. The results for the test patients were aggregated, and overall performance was evaluated based on all test predictions. Finally, we performed a SHAP analysis to identify the main driving features for each classifier (Rodríguez-Pérez and Bajorath, 2020).

### Code accessibility

The code described in the paper is freely available online at https://github.com/miriamguen/miriamguen-surface_eeg_seizure_analysis.git.

10.1523/ENEURO.0157-24.2024.d1 Extended Data 1 This item provides the latest version of the code zipped from our GitHub repository. Within this zipped folder is a README.md file that provides the instructions to run each analysis step presented in this manuscript. Download Extended Data 1, ZIP file.

## Results

In this study, we explored the use of a blind source separation approach on surface EEG data to extract seizure information and to identify the BifM of transitions into and out of seizures according to the TSD. We analyzed 1,177 seizures from 158 patients with focal epilepsy who were undergoing presurgical evaluation. Utilizing multichannel ictal EEG data, we applied a PCA–ICA preprocessing approach to separate sources within the ictal signals, resulting in a set of eight ICs for each seizure. The ICs were visually analyzed and labeled for clear transitions and their corresponding BifMs by two independent reviewers. We then used these labels to examine the interaction of the BifMs with various clinical factors. Additionally, to extend the applicability of our findings to a broader population, we used these labels as a gold standard to develop a scalable automated classification pipeline.

### Label validation

Two reviewers evaluated 1,177 seizures and a total of 9,416 ICs. The top part of Table 1 presents the number of ICs labeled with a clear transition and BifM by each reviewer and the number of ICs where both reviewers agreed on identifying a clear transition and the same BifM. Extended Data Table 1-2 contains the raw labels of both reviewers, which were used to create the summary table and perform the agreement analysis.

The overall agreement evaluation in both tasks is presented in Table 2, as evaluated using Cohen's kappa coefficient, which indicates strong agreement (Landis and Koch, 1977; McHugh, 2012). We conducted a permutation analysis to account for class imbalance in our data. The results demonstrate that inter-rater agreement in both tasks was significantly better than chance (*p* = 0.0001) using two different permutation approaches (Table 2).

**Table 2.** Component agreement statistics: the table below shows the inter-rater agreement statistics, including empirical and permutation analyses of kappa values

Category	ICs in the analysis	Empirical kappa	Mean kappa within-seizure permutation	Mean kappa overall permutation
All clear transition onset	9,416	0.658	0.198	−0.00014
All clear transition offset	9,416	0.641	0.210	−5.28 × 10^−6^
All labeled onset BifM	9,416	0.655	0.191	−9.14 × 10^−5^
All labeled offset BifM	9,416	0.628	0.198	4.60 × 10^−5^
Shared labeled BifM onset	1,132	0.881	0.659	7.39 × 10^−5^
Shared labeled BifM offset	948	0.840	0.673	−1.39 × 10^−5^

We conducted two permutation analyses to estimate the chance level for kappa values. In the first, we randomly permuted the reviewers’ labels overall. In the second, we permuted labels only within each seizure, maintaining the same number of ICs labeled by each reviewer per seizure. Each analysis included 10,000 permutations; the table presents the mean values. The within-seizure permutations yielded higher kappa values. However, all permutation results were below the empirical kappa value, indicating that the performance was significantly better than chance (*p* < 0.0001). BifM, bifurcation morphology.

A concern in surface EEG data is the low signal-to-noise ratio; in nonictal data, ICA is typically used to identify and remove noise sources. Consequently, several automated classifiers have been developed to distinguish between brain and noise sources. In this work, we used the mean value of two approaches: MARA (Winkler et al., 2011) and IClabel (Pion-Tonachini et al., 2019a), as briefly described in Materials and Methods, Feature extraction, and in Figure 2. Extended Data Figure 2-2*A* demonstrates that ICs labeled with a clear BifM correspond to a higher “brain” probability, indicating that the output of these classifiers can be used to identify ICs with clear seizure transition information. However, this is not specific to seizure data and can be high in brain sources that do not include clear seizure information, as shown in the control data and in the example in Extended Data Figure 1-1*D*.

We used a change-point detection algorithm to identify ICs with a clear objective transition. Extended Data Figure 1-1*B* descriptively demonstrates the proportion of segments with a detected change-point. The proportion was lowest for the control recordings, highest for the selected seizure ICs, and in between for the nonselected seizure time ICs. While this approach is not specific to ICs containing seizure transition information of a brain source, when combined with the brain score, it can be a good indicator of relevant seizure-related information.

### Detectability and BifM distribution at the seizure level

Table 1 presents the number of seizures with a clear BifM identified by each reviewer and by both reviewers, the mean number of ICs with a clear BifM per seizure, and the number of seizures with more than one BifM. Only ICs where both reviewers agreed on the BifM are defined as “clear” in the following section. Extended Data Figure 2-2*C* illustrates the selection flow for determining the seizure-level BifM used in cases where more than one IC included a clear BifM per seizure.

**Figure 3.**
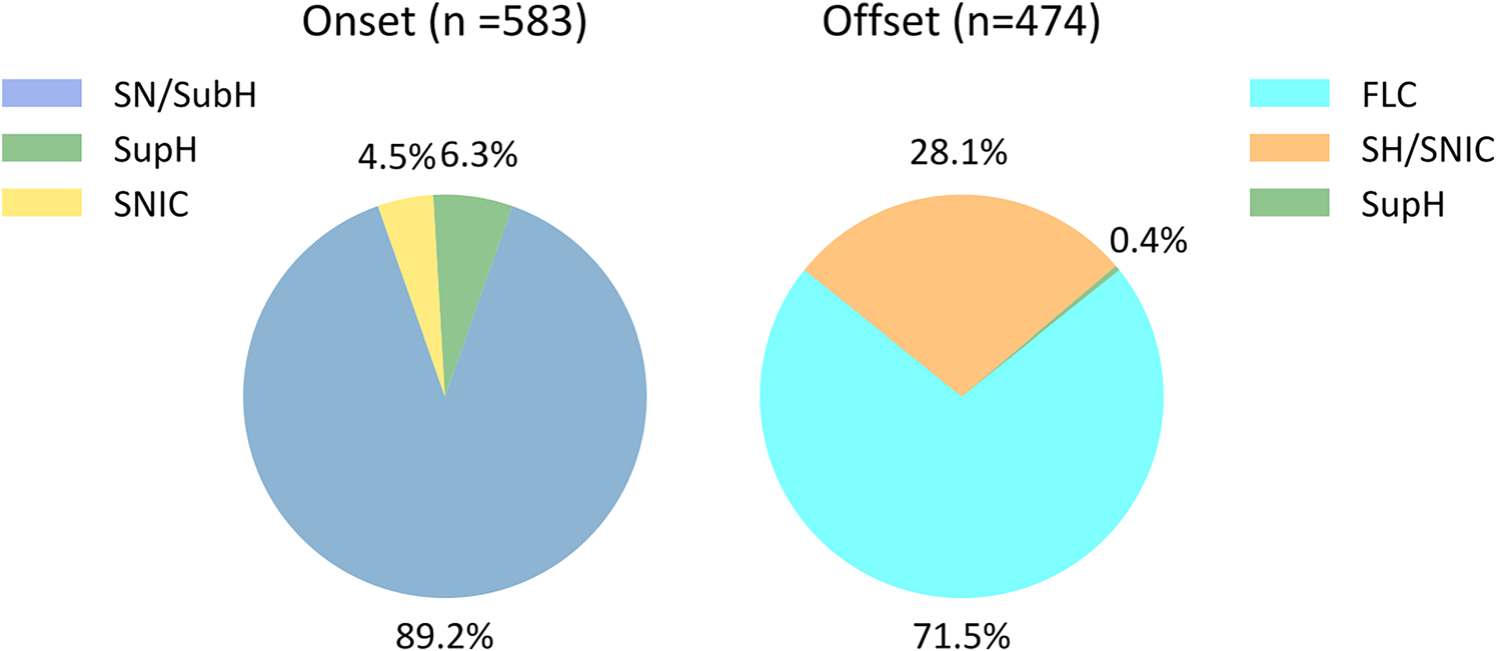
BifM distribution. The distribution of BifM in our data, encompassing all seizures marked by the onset or offset bifurcations, aligns broadly with previously reported intracranial data. However, the proportions of SN/SubH and FLC morphologies were higher in surface data. These morphologies do not necessitate specific patterns, such as ISI or amplitude changes. Additionally, the higher noise levels in surface data may obscure other morphologies, potentially leading to an overestimation of these patterns.

In 49.5 and 40.3% of seizures, we identified onset and offset BifMs that met our criteria. Onset and offset BifMs were not identified in 18 and 22 patients, respectively. The mean number of unique BifMs was higher in patients with more seizures (Extended Data Fig. 2-2*D*), suggesting that the number of observed seizures limits the number of observed BifMs. Detectability was better for seizures with a posterior SOZ and poorer for those with a frontal SOZ. Seizures with stronger clinical manifestations had a higher proportion of clear bifurcations, such as in FIAS and FBTCS, than in FAS or UC seizures (Extended Data Fig. 2-2*E*,*F*). Figure 3*A* demonstrates the overall distribution of onset and offset BifMs, showing that their rank aligns with previous findings (Saggio et al., 2020). Table 3 summarizes the onset and offset BifM pairs identified.

10.1523/ENEURO.0157-24.2024.t3-1 Table 3-1 The empirical dynamotype prevalence in the data is ordered by the mathematical complexity ranking, showing that low complexity is aligned with higher prevalence. Download Table 3-1, XLSX file.

**Table 3.** A summary of the onset and offset seizure BifM, including seizures with at least one (onset or offset) clear BifM

Onset/offset	FLC	SH/SNIC	SupH	Not clear	Clear	All
SN/SubH	241	84	1	194	326	520
SupH	13	6	0	18	19	37
SNIC	9	8	0	9	17	26
Not clear	76	35	1	0	112	112
Clear	263	98	1	221	362	583
All	339	133	2	221	474	695

Not all seizures with a clear onset included a clear offset and vice versa.

The prevalence of dynamotypes is hypothesized to be inversely related to their complexity. Previous work estimated this complexity using the canonical model, assuming hysteresis-loop bursting, i.e., bistability and feedback-based slow variables driving the onset and offset bifurcations Extended Data Table 3-1 summarizes the dynamotype count in our data, ordered by this ranking. Consistent with theoretical expectations, the most prevalent dynamotypes were of lower complexity. However, some exceptions were observed, which may be attributed to several factors limiting the application of the burster ranking to the data. These include the mathematical ranking derived for hysteresis-loop bursting dynamics, while other dynamics, such as slow-wave bursting, may also exist. Additionally, the FLC at the offset and SN/SubH at the onset lack specific features for identification, combined with the noise in surface EEG, which may lead to their overestimation in the visual analysis.

### Relationship with clinical factors

The comparisons that met the defined criteria are presented in Table 4, with the complete analysis results provided in Extended Data Tables 4-1 and 4-2. Robust findings were observed only in the comparisons of onset BifMs. While other trends were noted in the data, a larger cohort is necessary to draw definitive statistical conclusions.

**Table 4.** Onset comparisons in the Bayesian posterior analysis have a high pd and fall mostly outside the ROPE

Class	Parameter	Median	MAP	CI low	CI high	PD	ROPE
SupH	NREM1 – Awake	−0.042	−0.039	−0.092	−0.004	0.982	0.013
SupH	NREM2 – Awake	−0.038	−0.034	−0.084	−0.008	0.992	0.011
SNIC	NREM3 – Awake	−0.034	−0.022	−0.114	−0.003	0.980	0.012
SupH	FAS – FIAS	−0.052	−0.041	−0.146	−0.007	0.986	0.010
SupH	FBTCS – FIAS	−0.059	−0.041	−0.160	−0.014	0.999	0.001
SupH	FBTCS – UC	−0.085	−0.070	−0.200	−0.023	1.000	0.001
SupH	FAS – UC	−0.078	−0.066	−0.189	−0.016	0.994	0.004
SupH	Cortical dysplasia—Hippocampal sclerosis	0.056	0.045	0.009	0.143	0.993	0.009

The direction of the effect and effect size can be estimated by the median or MAP and the 95% CI range provided in the table. Extended Data Figure 4-1 provides a detailed and graphical explanation of the presented metrics. All the comparisons performed are provided in Extended Data Tables 4-1 for the onset and 4-2 for the offset.

Based on the defined criteria, at the patient level, seizures associated with cortical dysplasia had a higher proportion of SupH than those associated with hippocampal sclerosis. In a previous study, the SupH proportion was higher with older age (Saggio et al., 2020). Our data showed a similar trend that did not meet the defined criteria (Extended Data Table 4-1).

At the seizure level, no differences in BifM proportions were observed across the NREM stages. However, during wakefulness, there was a higher proportion of SupH BifMs compared with NREM1 and NREM2 and a higher proportion of SNIC BifMs compared with NREM3 (Table 4). Additionally, FIAS and UC seizures had a higher proportion of SupH BifMs than FBTCS and FAS seizures.

Extended Data Table 4-3 shows the relationship between vigilance and clinical classification, replicating previous findings in focal onset seizures (Herman et al., 2001). FBTCS, which occurs more frequently in NREM, had a lower proportion of SupH BifMs than FIAS and UC, possibly indicating a shared effect. In contrast, FAS seizures mostly begin during wakefulness, in which SupH BifMs are more common. FIAS seizures occur at similar rates during wakefulness, while UC seizures are less frequent. However, FAS seizures have a lower proportion of SupH BifMs compared with both FIAS and UC seizures. These observations suggest distinct connections between dynamical patterns, vigilance states, and the clinical manifestations of seizures.

### Automated classification

#### Automated classification of ICs for clear seizure transition

**Figure 4.**
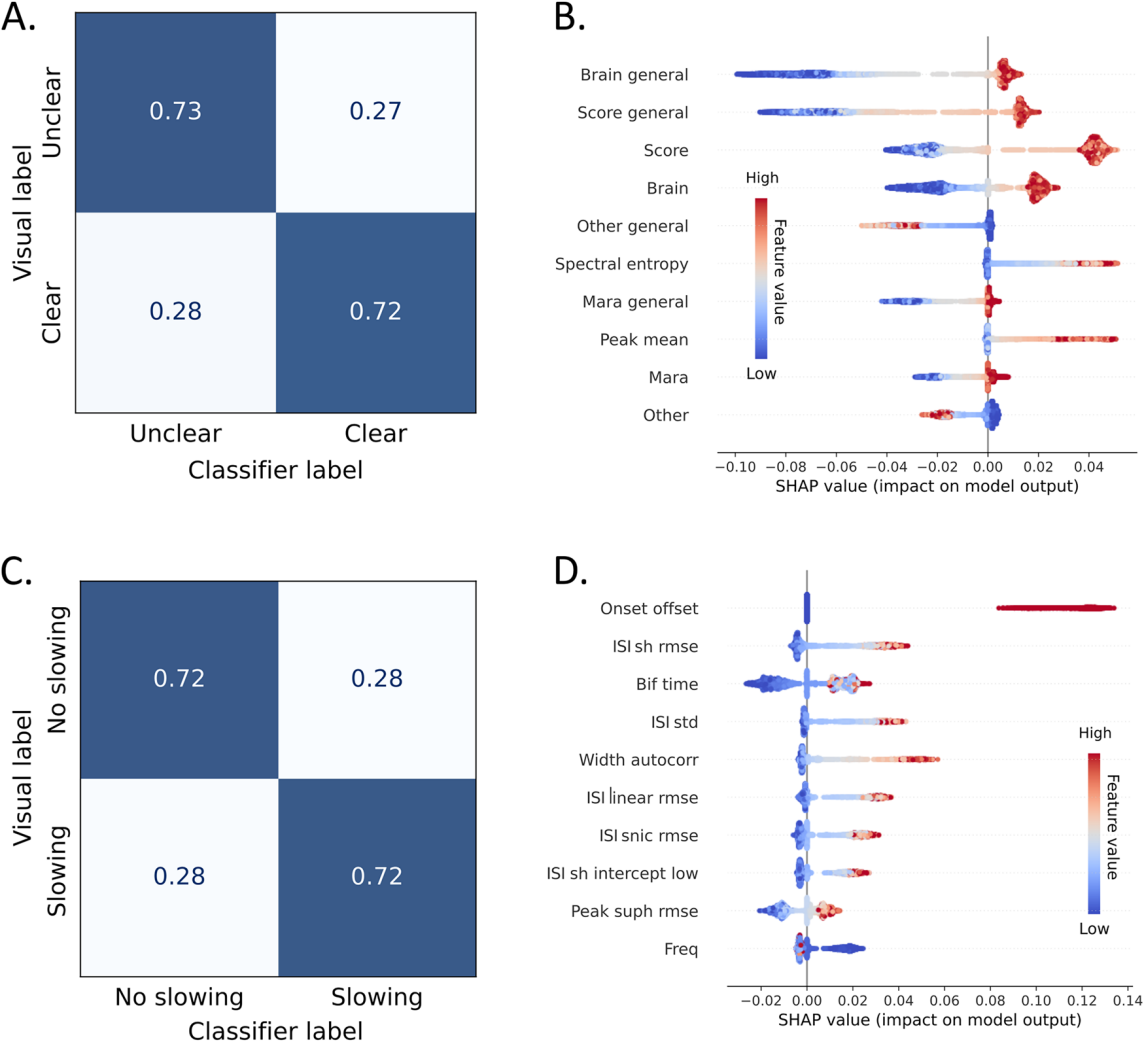
General classifier analytics. This figure presents the analysis of two classification tasks. The first classifier identifies components with a clear transition among all given components, while the second classifier identifies components exhibiting ISI slowing based on BifM. Both classifiers were trained using the same feature set and algorithm hyperparameters, with class weights adjusted differently to account for class imbalance and to achieve balanced performance. Each classifier was evaluated using a LOOCV approach. The confusion matrices (panels ***A*** and ***C***) display cumulative classification results on the test sets. To understand the classification process, feature contributions were analyzed using SHAP analysis (Rodríguez-Pérez and Bajorath, 2020), highlighting the most influential features of each classifier and their directional effects. ***A***, The confusion matrix for the transition detection task. ***B***, Dominant features for the transition detection task: high brain probability scores were associated with clear components, while high probability scores for undefined noise sources (“other”) indicate a lower likelihood of being seizure components. High spectral entropy, a feature previously used in seizure detection tasks (Boonyakitanont et al., 2020), supported positive decisions. High mean peak amplitude in the first 5 s of detected onset/offset was related to clear seizure transitions. ***C***, The confusion matrix for the ISI slowing classification task. ***D***, Top features for the ISI slowing classification task: a value indicating an offset increased the probability of ISI slowing due to the higher likelihood of this pattern occurring at offsets. Seizure length was positively correlated with ISI probability, although no significant trend was observed in multilevel analysis. Lower dominant frequency and higher standard deviation in ISIs were associated with ISI slowing. The ISI linear intercept estimate (higher CI limit) and lower fit quality (RMSE) to the ISI trend curves were positively related to slowing. This may be due to low error in cases where the fitted line was flat and the observation that slowing usually occurred in components with higher amplitudes, as suggested by the peak trend estimation relation.

We analyzed 9,416 ICs for seizure onset and offset to identify clear transitions, resulting in 18,832 examples; 1,103 had a clear onset, and 884 had a clear offset transition. In cases where feature extraction was unsuccessful (e.g., due to noise or insufficient peaks), we used empty values to ensure output continuity. We evaluated performance using the LOOCV paradigm for each classification task, estimating performance with BA and AUC-ROC. Figure 4*A* presents the normalized confusion matrix from the cumulative labels of the cross-validation process. Table 5 summarizes the performance results for all subjects, while Extended Data Table 5-1 details the results by patient.

10.1523/ENEURO.0157-24.2024.f4-1 Figure 4-1 The Bayesian measures used for evaluation. This figure provides an intuitive illustration of the measures presented in Table 4. We evaluated the difference between two posterior distributions of bifurcation morphologies, given the tested clinical factors compared to the null hypothesis. The reported measures used in this work are as follows, as suggested by (Makowski et al., 2019). Download Figure 4-1, TIF file.

10.1523/ENEURO.0157-24.2024.t4-1 Table 4-1 Onset bifurcation morphology distribution analysis. This table provides all the conditions compared in the current analysis. Download Table 4-1, XLSX file.

10.1523/ENEURO.0157-24.2024.t4-2 Table 4-2 Offset bifurcation morphology distribution analysis. This table provides all the conditions compared in the current analysis. Download Table 4-2, XLSX file.

10.1523/ENEURO.0157-24.2024.t4-3 Table 4-3 Seizure clinical classification crossed with sleep stages. This table provides a contingency matrix for 572 seizures analyzed for onset bifurcations (Saggio et al., 2017). In this table, we can observe that seizures undergoing generalization have a higher prevalence in deeper stages of sleep, as previously reported. Interestingly, we also see a high portion of generalization during REM. Download Table 4-3, XLSX file.

**Table 5.** Seizure transition classification performance metrics, as measured by LOOCV

	BA	ROC-AUC
Overall	0.727	0.810
Mean	0.719	0.813
STD	0.134	0.117
25%	0.657	0.764
Median	0.728	0.825
75%	0.795	0.888
Patients	158	148

The performance was evaluated in each training round and separately for each subject. Not all subjects had examples of both classes, limiting the ability to estimate the area under the receiver operating characteristic curve (AUC-ROC) for all individuals; the current table reports the mean, median, and standard deviation of the performance metrics over patients. The predicted probabilities for each test patient were saved through the cross-validation process, and the overall performance was estimated on all the joint prediction arrays to include all patients in the final estimate (“overall”). Extended Data Table 5-1 includes the classification results per patient.

10.1523/ENEURO.0157-24.2024.t5-1 Table 5-1 The full by-patient results of the seizure transition classification results and performance metrics, as measured by leave-one-subject-out cross-validation. Download Table 5-1, XLSX file.

Figure 4*B* presents the SHAP analysis results, highlighting the top 10 features influencing the model's decisions and the direction of the effect for each example. Eight of these features were derived from the MARA and IClabel classifiers. Low scores, indicating that the ICs are not from a brain source, led the classifier to an “unclear” label. In contrast, high scores showed a weak association with the “clear” label, suggesting that this information is necessary but insufficient for identifying clear brain-origin seizure transitions. Spectral entropy, a well-known feature used for seizure detection (Acharya et al., 2015), strongly identified an IC as seizure-related. A higher peak amplitude also contributed to classifying the data as containing seizure information.

#### Automated classification of ICs for ISI increase

For this stage, we used the ICs for which both reviewers agreed on the BifM during visual labeling. This included 1,103 onset ICs and 884 offset ICs (Table 1). Of these, 52 onset and 234 offset ICs showed an increase or decrease in ISI (from and to zero). The summary of the LOOCV results is presented in Table 6, with the detailed performance per patient provided in Extended Data Table 6-1. The overall confusion matrix reflecting performance is shown in Figure 4*C*, and the SHAP analysis results for this task can be seen in Figure 4*D*. The top feature was the onset/offset indicator, as ISI slowing was more prevalent at the offset in our data. Features quantifying ISI changes had the most significant impact on the classifier's decision. The width of the autocorrelation function emerged as a strong predictor of ISI slowing (Maturana et al., 2020).

**Table 6.** Seizure ISI slowing classification performance metrics, as measured by LOOCV

	BA	ROC-AUC
Overall	0.720	0.798
Mean	0.736	0.827
STD	0.231	0.216
25%	0.597	0.796
Median	0.774	0.886
75%	0.9	1
Patients	148	86

The current table includes the mean, median, and standard deviation of the performance metrics over patients and the overall performance, as described in Figure 4. Extended Data Table 6-1 includes the detailed data per patient that this summary table is based on.

10.1523/ENEURO.0157-24.2024.t6-1 Table 6-1 Full by-patient results of the ISI slowing classification results and performance metrics, as measured by leave-one-subject-out cross-validation. Download Table 6-1, XLSX file.

## Discussion

Recent studies employing a phenomenological modeling approach to develop the TSD have demonstrated its potential to provide clinically relevant insights. These computational models have been used to construct digital simulations representing the epileptogenic network in patients undergoing presurgical evaluation, improving surgical outcomes (An et al., 2019; Makhalova et al., 2022). Additionally, they have been utilized to optimize electrical stimulation for seizure abortion (Szuromi et al., 2023). Empirical findings from intracranial recordings (electrocorticography, ECoG) in humans have demonstrated that the prevalence of onset and offset bifurcations aligns with theoretical predictions (Saggio et al., 2020). Our study extended this classification to noninvasive EEG recordings, addressing several challenges.

The first challenge is the mixture of physiological and nonphysiological sources in surface EEG signals, requiring effective methods to isolate the information of interest. We addressed these challenges by employing a blind source separation approach, which decomposes the complex signals into ICs that are as statistically independent as possible (Makeig et al., 1995). We visually identified the ICs containing seizure transition information relevant to the TSD classification while accounting for the limitation of missing DC component in our recordings. This component distinguishes several onset and offset classes and has potential clinical relevance in predicting the response to seizure-aborting stimulation (Szuromi et al., 2023). This remains a limitation of our current analysis approach, which uses a high-pass filter that would eliminate a DC component if contained in the data. However, if DC information were available, this limitation could be addressed by applying a low-pass filter to the original signal to extract the DC components. Using the learned weights, the ICA transformation could then be applied to the low-frequency components, and this additional information could be incorporated into the classification.

The second challenge hinges on the essential difference between the nature of the recorded signals in the current and previous work. When recorded on the surface, the brain signal undergoes volume conduction and smearing as it traverses various mediums, such as the cerebrospinal fluid, dura mater, skull, and skin, before reaching the sensors (van den Broek et al., 1998). This process can alter the signal generated by neuronal tissue, affecting the morphology of the data. Additionally, the neuronal population required to detect activity in surface EEG is larger compared with intracranial recordings (Tao et al., 2007a,b), potentially reflecting different dynamical behaviors, such as seizure spread and propagation, that are not evident from a single electrode in invasive recordings. Conversely, the seizure onset may appear later compared with invasive recordings or may not be apparent in surface recording, depending on the depth of the ictal source (Koessler et al., 2010; Barborica et al., 2021). Thus, the dynamics captured in the surface data may reflect a larger-scale process of the epileptogenic network, and its interaction with the global brain state must be considered in the interpretation of our results.

Due to the inherent noise in the data, we applied strict labeling criteria, one of which required agreement between two independent reviewers to classify a transition as clear. This approach minimized the risk of misidentifying noise as seizure dynamics; for more detail, see Materials and Methods. Using these criteria, we identified clear transitions in 49.5% of the onsets and 40.3% of the offsets (Table 1). Clear onset and offset BifMs could not be identified in 18 and 22 patients, respectively. Detection rates were highest in occipital localizations and decreased toward frontal areas (Extended Data Fig. 2-2*E*). Additionally, detectability was better in more clinically pronounced seizures (Extended Data Fig. 2-2*F*), highlighting the influence of the involved neuronal tissue on the prominence of seizure activity in surface EEG (Tao et al., 2007b).

Our approach was based on the assumption that seizure information consists of high-amplitude synchronized activity. Consequently, seizure onsets characterized by low-voltage fast activity were potentially missed. However, we often observed onset transitions in ICs that began with low-amplitude fast activity and evolved into high-amplitude low–frequency activity. Previous work demonstrated a strong correlation between these interictal and surface IC patterns, with detectability on the surface primarily influenced by the depth of the seizure source (Barborica et al., 2021).

Overall, the BifM ranking aligned with previous findings (Saggio et al., 2020). However, the proportion of offset FLC was notably higher in our data, at 71.5% (Fig. 3) compared with the previously reported 53.6%, and onset SN/SubH was also higher, at 88% compared with 71.1%. Several factors may explain this discrepancy.

First, prior studies used intracranial recordings, which directly capture data from smaller neuronal populations at the seizure focus, reflecting finer spatial and temporal dynamics. In contrast, scalp EEG requires synchronized activity over larger brain regions to produce a detectable signal. Consequently, scalp EEG recordings reflect seizure activity at a different temporal and spatial scale, as detailed above. This inherent difference is likely a significant factor contributing to the discrepancies observed between our data and previous studies.

Second, the noise profile in surface EEG data differs, and despite preprocessing efforts, this noise could obscure features like amplitude or spike rate modulation. This may lead to the overclassification of BifMs that do not rely on distinct morphological features and underestimation of others. Third, from a clinical perspective, the proportion of BifMs (especially SupH) may be influenced by factors such as sleep stage, seizure type, or etiology (Table 4), making direct comparisons between studies challenging without accounting for these variables.

Previous work reported low numbers of SupH offset transitions, with 3% in recordings without DC components and 6% in recordings with them, consistent with TSD expectations (Saggio et al., 2020). In our data, this rate was even lower, at 0.4% (*n* = 3), which is possibly attributable to the distance between the seizure source and the recording electrodes. The TSD framework models seizure dynamics from a point source; however, the signal measured in surface EEG is indirect and undergoes transformations that may hinder the accurate detection of these patterns. This is further illustrated by a trend observed in seizure pathology. While SupH offsets were excluded from statistical modeling due to the small sample size, the onset analysis revealed a higher estimate of SupH BifMs in cortical dysplasia (a superficial source) compared with hippocampal sclerosis (a deeper source; Table 4)—a trend not found in intracranial data. Depending on the etiology, such considerations may also be relevant in intracranial data, particularly when comparing ECoG and depth electrodes.

At the onset transition, the observed amplitude increase in the SupH BifM in surface EEG may reflect propagation or be influenced by noise levels in the signal, rather than exclusively representing the dynamics of the neuronal population in the SOZ. In some cases, we observed amplitude increases related to propagation (Fig. 2*D*). While we attempted to differentiate these instances, some propagation events may have been misclassified as SupH. Typically, higher propagation is expected in seizures clinically classified as FBTCS. However, we observed lower SupH BifM rates in FBTCS compared with FIAS and UC seizures (Table 4), with rates nearly equivalent between FBTCS and FAS (Extended Data Table 4-1). These findings suggest that this morphology in surface EEG reflects dynamics beyond mere seizure propagation.

Several approaches could enhance our understanding of the surface and source signal differences. Simultaneous recordings have previously been employed to validate the correlation between source and surface activity, offering valuable insights into the spatial and temporal dynamics of seizures (Tao et al., 2007b; Koessler et al., 2010; Barborica et al., 2021). These approaches could be particularly useful for clarifying how SupH bifurcations manifest on the surface. Source imaging has shown significant utility in localizing epileptogenic foci and improving presurgical evaluations by integrating high-density surface EEG data with structural information to infer the origins of electrical activity (Michel et al., 2004; Plummer et al., 2008). This noninvasive technique could enhance the reconstruction of the SOZ time series, potentially providing a more accurate representation of the underlying neuronal dynamics.

The modeling framework underlying the development of the TSD is phenomenological, meaning it uses a mathematical model to describe patterns of neuronal activity based on the observed behavior rather than explicitly linking them to biological mechanisms. This approach simulates the transitions into and out of seizures, with a dynamical fixed point (FP) representing the resting state and a limit cycle (LC) representing the oscillatory activity during a seizure. These states capture the general patterns of brain activity during seizures, modeling their initiation and cessation without directly specifying the underlying cellular or network-level processes (Saggio et al., 2020).

The theoretical framework describes the seizure driving forces at three main time scales: fast, slow, and ultraslow, which drive the transitions into and out of a seizure. These transitions can occur through two mechanisms. First, when multiple attractors coexist, such as a stable FP and LC (bistability), the dynamics can be altered by introducing noise that shifts the system from one attractor to another. Noise has been previously demonstrated to drive transitions into seizures in neuronal networks, particularly in bistable systems, where random fluctuations can push the system from a stable resting state to an oscillatory seizure state (Deco et al., 2009). Second, changing the system's parameters can result in a sudden alteration of its dynamical map—for instance, by eliminating an FP and creating an LC—thereby causing the transition from a resting state to an oscillatory state through a bifurcation. Bifurcations have been widely proposed as a core mechanism underlying seizure onset, where small parameter changes in neuronal systems can lead to large-scale shifts in network dynamics (Da Lopes Silva et al., 2003). This bifurcation process is central to the theoretical foundation of the TSD framework (Saggio et al., 2020).

The TSD mathematical framework explicitly models the fast and slow variables. The fast variable is responsible for modeling the oscillatory activity of seizures and can transition from FP to LC dynamics. The slow variable is modeled with hysteresis-loop bursting, essentially using input from the fast subsystem to provide feedback to the fast variable, leading to the cessation of oscillations and providing an intrinsic mechanism to limit seizure duration (Izhikevich, 2000; Jirsa et al., 2014). A one-dimensional slow system can model this feedback loop, offering a simplified mathematical structure while retaining essential dynamical features. Thus, this framework forms the basis for the dynamotype ranking used in our analysis (Saggio et al., 2017).

This modeling approach was initially developed to simulate local seizure dynamics, focusing on the transitions into and out of seizures, where the slow variable's dynamics were primarily attributed to the tissue in the SOZ or its immediate surroundings (Jirsa et al., 2014). However, in seizures occurring within the broader context of the brain, interactions between the SOZ and other brain regions must be considered. These external regions can modulate the epileptogenic network, particularly in different sleep states (Moore et al., 2021). Shifting from local dynamics to a more comprehensive view that includes these interactions can deepen our understanding of seizure generation and propagation.

Models like the “virtual epileptic brain” (Jirsa et al., 2017) have been developed to simulate large-scale brain dynamics, focusing on the onset, propagation, and termination of seizures. These models use different excitability assumptions for the slow dynamics of the epileptogenic network, the propagation network, and the noninvolved zones. However, they primarily operate on short timescales and do not consider the ultraslow modulation of excitability. To better capture seizure dynamics over longer timescales, it could be beneficial to introduce an explicit ultraslow parameter that modulates the excitability in the different zones reflecting the ultraslow physiological processes. For example, this modulation could leverage established frameworks like the two-process model of sleep–wake regulation (Borbély, 2022) or utilize the empirically predicted seizure susceptibility cycles found in many patients with epilepsy (Karoly et al., 2021). This integration has the potential to shed light on the interaction between processes at multiple timescales driving seizure dynamics.

In this work, we began by applying the taxonomy to larger-scale brain signals, acknowledging the limitations discussed earlier. This allowed us to test consistency and uncover potential insights for future modeling at this scale. First, we analyzed the distribution of BifMs in the dataset and compared these with prior findings in local recordings. While the overall rank remained similar, the absolute proportions, particularly for SupH BifMs, differed, potentially due to noise, as noted earlier. In our analysis of the relationship between clinical factors, most findings were linked to changes in SupH proportions. However, given the limitations, these findings may be influenced by technical aspects rather than seizure dynamics. Conversely, changes in the ISIs are less likely to be affected by propagation artifacts, making them a more reliable target for investigation using this modality.

Our analysis reveals that the proportion of SNIC onsets was reduced during NREM3 sleep compared with the awake state (Table 4). Similar but weaker trends were noted when comparing wakefulness to NREM1 and NREM2 (Extended Data Table 4-1). In these comparisons, the SupH proportions were consistently higher during wakefulness, while the SN/SubH proportion increased during NREM sleep (Extended Data Table 4-1). Overall, SNIC and SupH onsets were more prevalent during wakefulness, aligning more closely with intracranial findings and theoretical expectations. NREM3, or slow-wave sleep (SWS), differs significantly from wakefulness in behavior and electrophysiology. In the context of seizures, research has shown that seizures are more likely to generalize during SWS (Herman et al., 2001), a finding reflected in our data (Extended Data Table 4-3).

Moreover, synchronized NREM sleep generally facilitates epileptogenic activity (Halász et al., 2019), while desynchronized REM sleep tends to be seizure-protective (Ng and Pavlova, 2013) and shows more localizable epileptogenic activity (McLeod et al., 2020). These findings demonstrate the importance of considering sleep stages in understanding seizure dynamics. In the context of the modeling approach, these observations could be viewed as the modulation of the ultraslow variable, leading to a change in excitability affecting the seizure rate and propagation or to the movement on the state space map affecting the observed BifMs.

Although our dataset includes many seizures (1,177), not all could be labeled for their BifM due to noise contamination (Table 1). Expanding this analysis to additional noninvasive datasets would increase the number of participants and the range of clinical conditions considered (e.g., generalized epilepsies). However, the visual classification process is labor-intensive and prone to inter-rater variability.

Previous studies have demonstrated significant inter-rater variability in EEG interpretation, highlighting the importance of multiple reviewers to improve diagnostic reliability (Grant et al., 2014; Zibrandtsen et al., 2019; Aanestad et al., 2024). Unlike previous studies focusing solely on time series analysis and long-term EEG recordings for rare seizures, we utilized data with prelabeled seizure onset and offset times. Within each seizure, we identified ICs containing seizure information from brain sources, relying on time series, IC topography, spectral profile, and automated source rating. We also conducted a training protocol with a large dataset of ICs and simulated data to enhance reviewer consistency. This protocol resulted in an inter-rater agreement that was significantly better than chance and is considered moderate to high (McHugh, 2012). Nonetheless, it is important to acknowledge that involving only two reviewers may limit the robustness of our findings. Future studies should consider including additional reviewers to enhance the reliability of EEG classifications.

Automating this process would enable the extension of this analysis to larger cohorts and a broader population. In this work, we used a baseline classification algorithm to examine the utility of features for identifying seizure ICs and classifying the ISI slowing (based on the limitations mentioned above). We demonstrated good cross-subject performance in both tasks. The top contributing features for identifying seizure information included brain score indicators (Winkler et al., 2011; Pion-Tonachini et al., 2019a) and entropy measures typically used for seizure detection (Boonyakitanont et al., 2020). For classifying ISI slowing, the top features were morphological characteristics and the width of autocorrelation function (Maturana et al., 2020). This classification framework could be further tested and improved by employing a more extensive dataset.

Current and previous work has focused primarily on the seizure onset and offset bifurcations. Automated detection tools, particularly change-point analysis (Truong et al., 2020) throughout a seizure, combined with automated classification, may enable the dynamics throughout the spatiotemporal evolution of seizures in empirical data to be quantified, providing additional input for modeling epileptogenic networks in patients. Moreover, by reducing the labeling workload, such automated classifiers could extend this analysis to a more extensive database, allowing seizure dynamic changes across a broader range of clinical conditions to be examined with greater statistical power, feeding back information and improving modeling approaches.

## Synthesis

Reviewing Editor: Viji Santhakumar, University of California Riverside Decisions are customarily a result of the Reviewing Editor and the peer reviewers coming together and discussing their recommendations until a consensus is reached. When revisions are invited, a fact-based synthesis statement explaining their decision and outlining what is needed to prepare a revision will be listed below. The following reviewer(s) agreed to reveal their identity: William Stacey, Marisa Saggio.

SYNTHESIS: This study presents a novel method for automated classification seizure dynamotypes and applies this classification to large seizure database with scalp EEG data. The ability to use the approach to analyze scalp EEG data has huge potential clinical relevance. The reviewers felt that these methods are useful and robust, and that the revised manuscript was much easier to follow. Several aspects of the analysis were assessed as cutting edge and new to this field. The revisions addressed most initial concerns. The reviewers found the the shifted focus towards slower timescales of great interest. However, this raised a few more points/clarifications are needed to improve the manuscript.

1. The statement that "First, prior studies were based on intracranial recordings, which capture data from a smaller neuronal population that may exhibit different dynamics." is much more important than implied here. This statement makes it appear that recording from smaller populations might be less robust. This statement should be expanded to state that scalp EEG requires very large regions of brain to produce a signal, and that is it common for the scalp not to show the seizure until after considerable delay, especially with deep seizures (which are the majority here). Thus, the scalp recordings are inherently biased to when the seizure has already spread to a large region of brain, rather than the dynamics of the seizure focus seen on iEEG. This is undoubtedly the most important reason for the difference.

2. Regarding feature extraction, lumping together time forward and time reversal signals is not justified when considering the underlying assumption of the mechanism (different bifurcations) generating these signals. Specifically, L 227-228 - To increase the size of the dataset for feature extraction, the authors time reverse offset events. This might not pose a problem when considering the supH bifurcation, which can occur with monostability and a slow-wave mechanism for the slow variables so that the post ictal and pre ictal phases are in fact comparable. However this bifurcation was excluded from the offset dataset because rare. For all other bifurcations considered, mixing onset and offset would further lump together bifurcations leading to two big groups depending on the BMs: (i) subH (onset), SN (onset) and FLC (offset) and (ii) SNIC (onset and offset) and SH (offset). Beside the lumping per se, what concerns me is that all these bifurcations (except the SNIC which can also occur with slow wave mechanism) are necessarily related to hysteresis phenomena so that the preictal state and the postictal one have different characteristics. In all onset cases, the preictal state gradually comes closer to the bifurcation point and crosses it, so the related signal can exhibit critical slowing down or other features related to the approach of the specific bifurcation destabilizing the resting state. On the other hand, when the system goes back to rest after a SH or FLC offset, because of bistability, the postictal state is a fixed point not involved in the bifurcation at all, so its related signal will not show any bifurcation-related feature. For this reason, transforming offsets in onset by time reversal, when hysteresis is involved, and lumping them together with real onset, will make interesting features computed from the non ictal segment (such as the autocorrelation function and its width) much less informative. If the this analysis is retained, the authors should discuss this point in conjunction with the role of the 'categorical feature' they use to identify differences between the time forward and time reversal groups (lines 284-285).

3. The discussion about the slower timescale sometimes mixes different levels of the modeling. This confusion may be removed by acknowledging that slower timescales include at least two groups of phenomena: (i) those acting on the timescale of ictal length (from now on 'slow'), responsible for seizure termination and reasonably relying on feedback from the fast subsystem and hysteresis (because seizures do usually terminate within a limited time unless something overrides these mechanisms and the consequence is status epilepticus) (ii) mechanisms leading to increased seizure propensity that act on an even slower timescales (from now on 'ultra-slow') for which we have not yet evidence in favor of one specific mechanism and slow-wave and hysteresis-loop mechanisms could also coexists. Some specific points:

3a. L 62 It is correct that the slow subsystem corresponds to variables that change on timescales slower than those of the fast subsystems. However, in the TSD framework, these slower processes are separated in a 'slow' component, explicitly modeled with the z variable dynamics, and an ultra-slow one that can affect seizure propensity by changing the excitability parameter or by affecting the map's parameters, thus moving the system in the map and that is discussed in the TSD framework but not explicitly modeled. All the examples reported in lines 63-67 belong to this latter ultra-slow component). It would be better to rephrase as 'slow subsystem corresponds to changes in brain state that occur over significantly longer timescales.' with something on the line of 'slow subsystem corresponds to the slow mechanisms that concur to seizure termination and changes in brain state that occur over significantly longer timescales and can modify the susceptibility to seizures.'.

3b. L 554 the virtual epileptic patient approach does make use of different assumptions for regions within and outside the SOZ by using different values for the excitability parameter of the slow dynamics z. What the authors are suggesting would be to add to the model an explicit ultra-slow modulation of the parameters (excitability in the slow dynamics or the fast parameters determining the location on the map) through coupling with models of the ultra-slow variables, such as the two process model for sleep/wake cycle or others. This direction very interesting. It may be useful to distinguish the slow and ultra-slow timescale to allow for a clearer discussion. In particular, see lines 599-603 where it would be more reasonable to hypothesize an (ultra-slow) slow-wave mechanism to influence seizure susceptibility and thus onset, through effects on the excitability and possibly also movement on the map and thus seizure class, but retain the hysteresis-loop slow mechanism for seizure offset - otherwise how would seizure terminate on a much shorter timescale than the proposed ultra-slow fluctuations if the offset mechanism doesn't rely on coupling from the fast one?

3c. L 61 as noted above, the fast subsystem represents fast neural activity - depending on the value of the excitability parameter (of the slow variable) it models 'healthy' brain regions or those in the SOZ

4. The discussion is inappropriately long, goes far beyond the scope of the paper and can be reduced by 50%. It should be focused just on the work at hand. For example several paragraphs which focuses on a specific model and limited findings: "this modeling approach was initially developed..." "From a chronobiological perspective..." "Incorporating long-term processes..." and 1.5 pages of editorial that does not seem to be related to this work and could be removed. Similarly, 2 pages expounding about sleep physiology is not directly relevant and can be removed.

5. A prior concern regarding having at least three reviewers has not been addressed. The authors should acknowledge this limitation and cite papers about the variability of reviewer agreement(example PMID: 35460472).

6. The section on "Modeling the interactions between BMs" has several paragraphs introducing important steps about model intercept priors, posterior distributions, and defining the null hypothesis with ROPE. While this is rigorous and a strength of the paper, it should also have a very brief explanation describing what each of those things are (high level description) because they will be unfamiliar to a broad readership.

7. The examples of onsets in 2-1 should have better description of how the chosen bifurcations were chosen. In particular, there should be a description of why a particular Bifurcation was chosen (e.g. a, b do not explain why they are SN/SubH)

8. L 156-163 The authors should consider recalling the features of the six bifurcations they are looking for (maybe with a table). If they don't want to go into the details of each bifurcation, they should as a minimum briefly recap what they are looking for - six bifurcation types in principle - and that the BM features that can be used to distinguish them are three: presence/absence of DC shift, specific trends in the frequency or in the amplitude. Otherwise the rest may be obscure for a reader who is not familiar with the framework.

9. Line 52 'comprehensive' is likely too strong. TSD relies on assumptions (eg. 2D systems)

## Author Response

Synthesis Statement for Author (Required):

SYNTHESIS: This study presents a novel method for automated classification seizure dynamotypes and applies this classification to large seizure database with scalp EEG data. The ability to use the approach to analyze scalp EEG data has huge potential clinical relevance. The reviewers felt that these methods are useful and robust, and that the revised manuscript was much easier to follow. Several aspects of the analysis were assessed as cutting edge and new to this field. The revisions addressed most initial concerns. The reviewers found the shifted focus towards slower timescales of great interest. However, this raised a few more points/clarifications are needed to improve the manuscript.

Comment 1 The statement that "First, prior studies were based on intracranial recordings, which capture data from a smaller neuronal population that may exhibit different dynamics." is much more important than implied here. This statement makes it appear that recording from smaller populations might be less robust. This statement should be expanded to state that scalp EEG requires very large regions of brain to produce a signal, and that is it common for the scalp not to show the seizure until after considerable delay, especially with deep seizures (which are the majority here). Thus, the scalp recordings are inherently biased to when the seizure has already spread to a large region of brain, rather than the dynamics of the seizure focus seen on iEEG. This is undoubtedly the most important reason for the difference.

Response 1 We appreciate the reviewer's comment highlighting the significant differences between intracranial and scalp EEG recordings. Our intention was not to imply that recordings from smaller neuronal populations are less robust. Rather, we aimed to emphasize that scalp EEG captures brain dynamics at a broader and coarser scale compared to intracranial EEG.

As the reviewer correctly points out, scalp EEG requires synchronized activity over larger regions of the brain to produce a detectable signal. Consequently, scalp EEG recordings reflect seizure activity at different temporal and spatial scales, as discussed in the preceding paragraphs in the Discussion. This inherent difference is likely to account for the discrepancies observed between our data and previous studies that utilized intracranial recordings.

To emphasize this important point, we have revised the relevant section in the manuscript to clarify these distinctions. The updated paragraph now reads: "First, prior studies used intracranial recordings, which directly capture data from smaller neuronal populations at the seizure focus, reflecting finer spatial and temporal dynamics. In contrast, scalp EEG requires synchronized activity over larger brain regions to produce a detectable signal. Consequently, scalp EEG recordings reflect seizure activity at a different temporal and spatial scale, as detailed above. This inherent difference is likely a significant factor contributing to the discrepancies observed between our data and previous studies." We hope that this revision addresses the reviewer's concerns and clarifies the differences between intracranial and scalp EEG recordings in our study.

Comment 2 Regarding feature extraction, lumping together time forward and time reversal signals is not justified when considering the underlying assumption of the mechanism (different bifurcations) generating these signals. Specifically, L 227-228 - To increase the size of the dataset for feature extraction, the authors time reverse offset events. This might not pose a problem when considering the SupH bifurcation, which can occur with monostability and a slow-wave mechanism for the slow variables so that the post ictal and pre ictal phases are in fact comparable. However, this bifurcation was excluded from the offset dataset because rare. For all other bifurcations considered, mixing onset and offset would further lump together bifurcations leading to two big groups depending on the BMs: (i) SubH (onset), SN (onset) and FLC (offset) and (ii) SNIC (onset and offset) and SH (offset). Beside the lumping per se, what concerns me is that all these bifurcations (except the SNIC which can also occur with slow wave mechanism) are necessarily related to hysteresis phenomena so that the preictal state and the post-ictal one have different characteristics. In all onset cases, the preictal state gradually comes closer to the bifurcation point and crosses it, so the related signal can exhibit critical slowing down or other features related to the approach of the specific bifurcation destabilizing the resting state. On the other hand, when the system goes back to rest after a SH or FLC offset, because of bistability, the post-ictal state is a fixed point not involved in the bifurcation at all, so its related signal will not show any bifurcation-related feature. For this reason, transforming offsets in onset by time reversal, when hysteresis is involved, and lumping them together with real onset, will make interesting features computed from the non ictal segment (such as the autocorrelation function and its width) much less informative. If the this analysis is retained, the authors should discuss this point in conjunction with the role of the 'categorical feature' they use to identify differences between the time forward and time reversal groups (lines 284-285).

Response 2 We appreciate the reviewers' comments and understand the concern regarding the potential lumping of the onset and offset transitions. We want to emphasize that the goal of this step was to increase the size of the training dataset available for the ISI classification task, which does not directly model the onset and offset mechanisms but focuses on the morphology of seizure onset and offset.

First, it is important to clarify that the morphological features we extract-such as inter-spike interval (ISI) changes and the width of the autocorrelation function-are derived from the ictal segment (the first or last five seconds of the seizure) rather than from the peri-ictal or post-ictal segments. We acknowledge that pre-ictal and post-ictal states may exhibit different characteristics, especially in bifurcations involving hysteresis. However, our approach was not intended to model these phenomena directly. We agree that evaluating feature dynamics (e.g., critical slowing) during peri-ictal phases at different time scales is an interesting avenue for future research.

Still, it is reasonable to expect inherent differences between seizure onset and offset. Recognizing this, we introduced a categorical indicator feature into the random forest ensemble classifier. This feature allows the model to learn distinct decision flows for onset and offset transitions, preserving the distinction between these two groups in the classification task. At the same time, it enables the classifier to leverage common patterns shared between onset and offset cases. Model diagnostics (SHAP analysis) demonstrate that this categorical feature is the most strongly ranked, indicating significant inherent differences between the cases. Despite this, modeling onset and offset separately yielded significantly lower performance, which is why we decided to focus on the joint classification approach. Nonetheless, the provided codebase can be used to train the model separately on onset or offset, which would be advisable with a larger labeled dataset in the future.

Regarding the width of the autocorrelation function, although an informative feature in the current classification task, prior studies indicate that signs of critical slowing down are reflected in the feature's dynamics over longer time scales (the rising phase) rather than in absolute values. In our analysis, we focus on a snapshot of five ictal seconds close to the transition without quantifying the temporal trend. Therefore, the relationship with critical slowing down is limited here. Considering this, we have retracted the text relating this feature to the critical slowing in the results and discussion sections.

To better clarify these points, we have revised the relevant text in the "Automated Classification" methods sections to provide further explanation of the role of the categorical feature and how it separates onset and offset signals during classification. We additionally clarified in the "Feature extraction" section that the morphological features extracted were extracted from ictal rather than non-ictal sections.

Comment 3 The discussion about the slower timescale sometimes mixes different levels of the modeling. This confusion may be removed by acknowledging that slower timescales include at least two groups of phenomena: (i) those acting on the timescale of ictal length (from now on 'slow'), responsible for seizure termination and reasonably relying on feedback from the fast subsystem and hysteresis (because seizures do usually terminate within a limited time unless something overrides these mechanisms and the consequence is status epilepticus) (ii) mechanisms leading to increased seizure propensity that act on an even slower timescales (from now on 'ultra-slow') for which we have not yet evidence in favor of one specific mechanism and slow-wave and hysteresis-loop mechanisms could also coexists.

Some specific points:

3a. L 62 It is correct that the slow subsystem corresponds to variables that change on timescales slower than those of the fast subsystems. However, in the TSD framework, these slower processes are separated in a 'slow' component, explicitly modeled with the z variable dynamics, and an ultra-slow one that can affect seizure propensity by changing the excitability parameter or by affecting the map's parameters, thus moving the system in the map and that is discussed in the TSD framework but not explicitly modeled. All the examples reported in lines 63-67 belong to this latter ultra-slow component). It would be better to rephrase as 'slow subsystem corresponds to changes in brain state that occur over significantly longer timescales.' with something on the line of 'slow subsystem corresponds to the slow mechanisms that concur to seizure termination and changes in brain state that occur over significantly longer timescales and can modify the susceptibility to seizures.'.

3b. L 554, the virtual epileptic patient approach does make use of different assumptions for regions within and outside the SOZ by using different values for the excitability parameter of the slow dynamics z. What the authors are suggesting would be to add to the model an explicit ultra-slow modulation of the parameters (excitability in the slow dynamics or the fast parameters determining the location on the map) through coupling with models of the ultra-slow variables, such as the two process model for sleep/wake cycle or others. This direction is very interesting. It may be useful to distinguish the slow and ultra-slow timescale to allow for a clearer discussion. In particular, see lines 599-603 where it would be more reasonable to hypothesize an (ultra-slow) slow-wave mechanism to influence seizure susceptibility and thus onset, through effects on the excitability and possibly also movement on the map and thus seizure class, but retain the hysteresis-loop slow mechanism for seizure offset - otherwise how would seizure terminate on a much shorter timescale than the proposed ultra-slow fluctuations if the offset mechanism doesn't rely on coupling from the fast one? 3c. L 61, as noted above, the fast subsystem represents fast neural activity - depending on the value of the excitability parameter (of the slow variable) it models 'healthy' brain regions or those in the SOZ Response 3 We thank the reviewers for this educational comment, and we agree that distinguishing between the slow and ultra-slow timescales is crucial for clarifying our modeling discussion. We have addressed the specific points as follows:

Regarding Points 3a+c:

We have revised the Introduction to acknowledge the separation between the fast, slow, and ultra-slow subsystems within the TSD framework. Specifically, we clarified that the slow subsystem corresponds to mechanisms acting on the timescale of ictal length, responsible for seizure termination through feedback from the fast subsystem and hysteresis. The ultra-slow subsystem includes mechanisms that act on longer timescales, influencing seizure propensity by modifying excitability or other parameters. The revised introduction section now reads:

Revised related introduction section: "The TSD approach describes seizure transitions using several time scales: a fast subsystem that intermittently generates seizure activity; a slow subsystem that modulates the fast system's parameters, facilitating mainly seizure termination; and an ultra-slow subsystem that can effect seizure propensity (Saggio et al., 2020). From this modeling perspective, the fast subsystem represents the seizure onset zone (SOZ); the slow subsystem represents reactive processes limiting seizure duration; and the ultra-slow subsystem corresponds to changes in brain state that occur over significantly longer timescales. The last, which is not explicitly modeled in the TSD framework, may include sleep stages (Herman et al., 2001), as well as circadian or longer physiological rhythms, which have been shown to affect seizure propensity (Karoly et al., 2021). Sleep stages, particularly wake, NREM, and REM, represent inherently different dynamical states with different tendencies toward epileptic events (Ng and Pavlova, 2013; Halász et al., 2019), making them a significant target for investigation." Regarding Point 3b:

We have updated the Discussion to distinguish clearly between the slow and ultra-slow timescales, allowing for a more coherent discussion of seizure dynamics. We acknowledge that the "virtual epileptic brain" approach incorporates different excitability parameters for regions within and outside the SOZ. We suggest enhancing these models by introducing an explicit ultra-slow modulation of parameters through coupling with fundamental models related to the sleep-wake cycle or empirical observations of fluctuations in seizure propensity recently described. The revised Discussion section now reads: "The theoretical framework describes the seizure driving forces at three main time scales: fast, slow, and ultra-slow, which drive the transitions into and out of a seizure. These transitions can occur through two mechanisms. First, when multiple attractors coexist, such as a stable FP and LC (bistability), the dynamics can be altered by introducing noise that shifts the system from one attractor to another. Noise has been previously demonstrated to drive transitions into seizures in neuronal networks, particularly in bi-stable systems, where random fluctuations can push the system from a stable resting state to an oscillatory seizure state (Deco et al., 2009). Second, changing the system's parameters can result in a sudden alteration of its dynamical map-for instance, by eliminating an FP and creating an LC-thereby causing the transition from a resting state to an oscillatory state through a bifurcation. Bifurcations have been widely proposed as a core mechanism underlying seizure onset, where small parameter changes in neuronal systems can lead to large-scale shifts in network dynamics (Da Lopes Silva et al., 2003). This bifurcation process is central to the theoretical foundation of the TSD framework (Saggio et al., 2020).

The TSD mathematical framework explicitly models the fast variable and slow variables. The fast variable is responsible for modeling the oscillatory activity of seizures and can transition from FP to LC dynamics. The slow variable is modeled with hysteresis-loop bursting, essentially using input from the fast subsystem to provide feedback to the fast variable, leading to the cessation of oscillations and providing an intrinsic mechanism to limit seizure duration (Izhikevich, 2000; Jirsa et al., 2014). A one-dimensional slow system can model this feedback loop, offering a simplified mathematical structure while retaining essential dynamical features. Thus, this framework forms the basis for the dynamotype ranking used in our analysis (Saggio et al., 2017).

This modeling approach was initially developed to simulate local seizure dynamics, focusing on the transitions into and out of seizures, where the slow variable's dynamics were primarily attributed to the tissue in the SOZ or its immediate surroundings (Jirsa et al., 2014). However, in seizures occurring within the broader context of the brain, interactions between the SOZ and other brain regions must be considered. These external regions can modulate the epileptogenic network, particularly in different sleep states (Moore et al., 2021). Shifting from local dynamics to a more comprehensive view that includes these interactions can deepen our understanding of seizure generation and propagation.

Models like the "virtual epileptic brain" (Jirsa et al., 2017) have been developed to simulate large-scale brain dynamics, focusing on the onset, propagation, and termination of seizures. These models use different excitability assumptions for the slow dynamics of the epileptogenic network, the propagation network, and the noninvolved zones. However, they primarily operate on short timescales and do not consider the ultra-slow modulation of excitability. To better capture seizure dynamics over longer timescales, it could be beneficial to introduce an explicit ultra-slow parameter that modulates the excitability in the different zones reflecting the ultra-slow physiological processes. For example, this modulation could leverage established frameworks like the two-process model of sleep-wake regulation (Borbély, 2022) or utilize the empirically predicted seizure susceptibility cycles found in many patients with epilepsy (Karoly et al., 2021). This integration has the potential to shed light on the interaction between processes at multiple timescales driving seizure dynamics.

In this work, we began by applying the taxonomy to larger-scale brain signals, acknowledging the limitations discussed earlier. This allowed us to test consistency and uncover potential insights for future modeling at this scale. First, we analyzed the distribution of BifMs in the dataset and compared these to prior findings in local recordings. While the overall rank remained similar, the absolute proportions, particularly for SupH BifMs, differed, potentially due to noise, as noted earlier. In our analysis of the relationship between clinical factors, most findings were linked to changes in SupH proportions. However, given the limitations, these findings may be influenced by technical aspects rather than seizure dynamics. Conversely, changes in the ISIs are less likely to be affected by propagation artifacts, making them a more reliable target for investigation using this modality.

Our analysis reveals that the proportion of SNIC onsets was reduced during NREM3 sleep compared to the awake state (Table 4). Similar but weaker trends were noted when comparing wakefulness to NREM1 and NREM2 (Table 4-1). In these comparisons, the SupH proportions were consistently higher during wakefulness, while the SN/SubH proportion increased during NREM sleep (Table 4-1). Overall, SNIC and SupH onsets were more prevalent during wakefulness, aligning more closely with intracranial findings and theoretical expectations. NREM3, or slow-wave sleep (SWS), differs significantly from wakefulness in behavior and electrophysiology. In the context of seizures, research has shown that seizures are more likely to generalize during SWS (Herman et al., 2001), a finding reflected in our data (Table 4-3).

Moreover, synchronized NREM sleep generally facilitates epileptogenic activity (Halász et al., 2019), while desynchronized REM sleep tends to be seizure-protective (Ng and Pavlova, 2013) and shows more localizable epileptogenic activity (McLeod et al., 2020). These findings demonstrate the importance of considering sleep stages in the understanding of seizure dynamics. In the context of the modeling approach, these observations could be viewed as the modulation of the ultra-slow variable, leading to a change in excitability affecting seizure rate and propagation or to the movement on the state space map affecting the observed BifMs." Comment 4 The discussion is inappropriately long, goes far beyond the scope of the paper and can be reduced by 50%. It should be focused just on the work at hand. For example several paragraphs which focuses on a specific model and limited findings: "this modeling approach was initially developed..." "From a chronobiological perspective..." "Incorporating long-term processes..." and 1.5 pages of editorial that does not seem to be related to this work and could be removed. Similarly, 2 pages expounding about sleep physiology is not directly relevant and can be removed.

Response 4:

We appreciate the reviewer's feedback regarding the length and focus of the Discussion section. In response to this comment, we have significantly reduced this section, ensuring it is more focused on the work at hand. Specifically, we have removed several paragraphs that extended beyond the scope of the paper, as described below:

We removed most of the content in the referred paragraphs that start with: "This modeling approach was initially developed...", "From a chronobiological perspective...", and "Incorporating long-term processes...", keeping only points related to the discussion specified in Comment 3.

We removed the discussion on sleep physiology, which was not essential to understanding our results. We have retained the key points related to seizure rate and propagation in different sleep stages, as well as the findings of different bifurcation distributions in these stages. These aspects are directly relevant to our study, as they may reflect changes in excitability and movement on the state space map, which are important for understanding the dynamics of the ultra-slow variable.

Some additions were made to the Discussion in response to other reviewers' comments, particularly those requesting clarification on specific points (e.g., Comments 1, 3, 5). We believe these additions are important and relevant to the discussion of our findings, however the net reduction in the discussion length that is lower than 50%, nonetheless we feel that the current discussion is more focoused on our findings as directed by the comments in this review.

Comment 5 A prior concern regarding having at least three reviewers has not been addressed. The authors should acknowledge this limitation and cite papers about the variability of reviewer agreement (example PMID: 35460472).

Response 5 We acknowledge that having only two reviewers is a limitation of the present work. While the paper associated with the provided PMID (35460472) addresses a different topic, we have cited recent studies that discuss inter-rater variability in EEG interpretation (Grant et al., 2014; Zibrandtsen et al., 2019; Aanestad et al., 2024). These studies highlight significant variability among reviewers in EEG interpretation, emphasizing the need for multiple reviewers to improve diagnostic reliability.

In our study, although only two reviewers performed the visual labeling, we implemented several measures to enhance consistency and reliability. The seizure onset and offset times were already labeled in the dataset, allowing our analysis to focus directly on the ictal and peri-ictal data points. The primary task involved identifying independent components (ICs) containing seizure information originating from a brain source. We relied not only on the time series but also on the IC topography, spectral profile, and automated source rating. Additionally, we conducted a separate training protocol using a large dataset of independent components to improve the reviewers' ability to identify brain sources.

The second task involved classifying the Bifurcation Morphologies (BifMs) using clear criteria (e.g., ISI trend, amplitude increase) within a limited time window from the labeled transition, designed to guide the reviewers toward consistent information.

As observed, the inter-rater reliability between the two reviewers was significantly higher than the chance level for both tasks, with kappa values indicating moderate to high agreement rates (McHugh, 2012). Nonetheless, we acknowledge that involving additional reviewers could enhance the robustness of the classifications. We recommend that future studies consider involving more reviewers to improve diagnostic reliability.

The following paragraph was added to the Discussion section to emphasize this: "Previous studies have demonstrated significant inter-rater variability in EEG interpretation, highlighting the importance of multiple reviewers to improve diagnostic reliability (Grant et al., 2014; Zibrandtsen et al., 2019; Aanestad et al., 2024). Unlike previous studies focusing solely on time series analysis and long-term EEG recordings for rare seizures, we utilized data with pre-labeled seizure onset and offset times. Within each seizure, we identified independent components (ICs) containing seizure information from brain sources, relying on time series, IC topography, spectral profile, and automated source rating. We also conducted a training protocol with a large dataset of ICs and simulated data to enhance reviewer consistency. This protocol resulted in an inter-rater agreement that was significantly better than chance and is considered moderate to high (McHugh, 2012). Nonetheless, it is important to acknowledge that involving only two reviewers may limit the robustness of our findings. Future studies should consider including additional reviewers to enhance the reliability of EEG classifications." Comment 6 The section on "Modeling the interactions between BMs" has several paragraphs introducing important steps about model intercept priors, posterior distributions, and defining the null hypothesis with ROPE. While this is rigorous and a strength of the paper, it should also have a very brief explanation describing what each of those things are (high level description) because they will be unfamiliar to a broad readership.

Response 6 We have edited the paragraph to include concise descriptions of key concepts such as model interception priors, posterior distributions, and the use of the Region of Practical Equivalence (ROPE) in defining the null hypothesis. We believe these additions enhance understanding while maintaining the rigor of our analysis. The revised paragraphs are as follows: "To analyze the relationship between BifMs and clinical factors at the patient and the seizure levels, we used the BRMS package (Bürkner, 2017) in R, which implements Bayesian multilevel models. These statistical models allow us to account for data hierarchies and repeated measures within a patient. Moreover, Bayesian models provide a probabilistic framework for statistical inference, incorporating prior knowledge and the observed data to estimate the parameters of interest.

Patient-level effects included age, gender, the lobe of the epileptogenic zone, and etiology. We treated the patient as a random effect for vigilance, seizure length, and classification to account for repeated measures within subjects. We included all three BifMs-SN/SubH, SNIC, and SupH-in a categorical logistic regression model for the onset. However, we encountered only three seizures with a SupH BifM for the offset. Consequently, we created a binomial model that included only SH/SNIC and FLC BifMs, excluding seizures with SupH BifM from the analysis.

In Bayesian statistics, a prior represents our initial belief about the parameters. Here, the intercept priors reflect our expectations about the BifM distribution. By initializing the model intercepts with the overall bifurcation distribution, we effectively assume a particular and similar BifM distribution across the clinical factors. Consequently, more robust evidence is required to deviate from this initial distribution during the model fitting process. This approach helps prevent overestimating effects due to random variations and makes our conclusions more robust.

The model distribution parameters were estimated using Monte-Carlo-Markov-Chain (MCMC). MCMC is a computational algorithm used to approximate the posterior distribution when it is difficult to calculate directly. After considering the observed data, the posterior distribution represents our updated beliefs about the parameters. For each model, we used six chains with 6,000 iterations each; the first 2,000 iterations were considered a warm-up (or burn-in period) and were excluded from the final posterior estimation to ensure that the chains had reached a stable distribution.

For adequate model fitting, we evaluated the fit quality in two ways. First, we performed a posterior predictive check (PP check) by simulating the overall posterior distribution and confirming that it accurately represented the observed data. This involves generating and comparing data from the model to the actual data to assess how well the model captures the underlying patterns. Second, we assessed the R ^ statistic, which measures the convergence between and within chains; an R ^ value greater than 1 indicates poor mixing and unreliable posterior estimates (Vehtari et al., 2021); in our models R ^ was equal to 1.

The Bayesian modeling approach allowed us to estimate posterior distributions under two clinical conditions, marginalized across all other factors, and to analyze the difference distribution to estimate the direction and effect size. For a centrality measure, we report the median and maximum a posteriori (MAP) of the difference distribution function and the credible interval (CI), which is equivalent to the frequentist confidence interval and provides a range within which the parameter value lies with a 0.95 probability (Figure 4-1A). To estimate the magnitude of the effect, we use the probability of direction (pd) (Figure 4-1B). The pd is mathematically defined as the proportion of the posterior distribution on the side of the median, effectively quantifying the certainty about the direction of an effect. It ranges between 0.5 (maximum uncertainty) and 1 (maximum certainty). A pd larger than 0.97 is considered to be a strong effect (Makowski et al., 2019).

We also used the full region of practical equivalence (ROPE) to define the null hypothesis over a range of values considered negligible or too small to be practically important. The ROPE approach allows us to determine whether an effect is not only strong but also if it is practically meaningful. This measure quantifies the percentage of the posterior distribution within the ROPE range (Figure 4-1C). In this study, we defined a 10% change in the minority class as the range of practical equivalence. A ROPE larger than 0.97 indicates strong support for the effect being practically negligible, while a ROPE lower than 0.03 indicates a meaningful effect size (Makowski et al., 2019)." Comment 7 The examples of onsets in 2-1 should have a better description of how the chosen bifurcations were chosen. In particular, there should be a description of why a particular Bifurcation was chosen (e.g. a, b does not explain why they are SN/SubH) Response 7 The legend of this figure was updated to describe the features used to determine the bifurcation types. We hope these revised legends provide a clearer explanation of why each example was classified as an SN/SubH bifurcation morphology. The revised labels are as follows:

Example of an SN/SubH bifurcation morphology (BifM) at the seizure annotation time. In this Independent Component (IC) time series, both the amplitude and the inter-spike interval (ISI) remain relatively constant during the transition into the seizure. There are no significant trends of increasing or decreasing amplitude or ISI. The absence of clear trends in these features led to the classification of this seizure onset as an SN/SubH BifM. This is the most prevalent onset type classified in our recordings.

Another example of SN/SubH BifM showing seizure onset activity. In this case, seizure activity with constant amplitude and rate begins approximately four seconds after the labeled seizure onset time, which is within the allowable range for classification. During the transition, the amplitude remains steady, and the ISI does not exhibit any systematic increase or decrease. These consistent features in amplitude and ISI are characteristic of SN/SubH BifMs. The discrepancy between the labeled seizure onset time and the actual component transition timing may be due to differences between electrographic and clinical seizure onset times or because the IC represents activity from a propagation zone rather than the seizure focus.

Comment 8 L 156-163 The authors should consider recalling the features of the six bifurcations they are looking for (maybe with a table). If they don't want to go into the details of each bifurcation, they should as a minimum briefly recap what they are looking for - six bifurcation types in principle - and that the BM features that can be used to distinguish them are three: presence/absence of DC shift, specific trends in the frequency or in the amplitude. Otherwise, the rest may be obscure for a reader who is not familiar with the framework.

Response 8 We appreciate the reviewer's suggestion to provide a brief recap of the six bifurcation morphologies (BifMs) and the key features used to distinguish them. We agree that including this information will make our work more accessible to readers who are not familiar with the framework.

To address this, we have added a concise description of the six BifMs. Moreover, these features are detailed in Figure 2-C, together with the visual BifM examples; we thus added a reference to this figure at this location. The revised paragraph is as follows: "Reviewers were also trained to identify seizure onset and offset bifurcation morphologies (BifMs) using empirical and simulation examples from previous work (Saggio et al., 2020). This classification was based on specific trends in frequency (inter-spike intervals) and amplitude changes. There are six BifMs in total-three onset types (SupH, SNIC, SN/SubH) and three offset types (SupH, SNIC/SH, FLC). SupH onsets show an amplitude increase from zero, while SupH offsets show amplitude reduction to zero; an increase in spike frequency characterizes SNIC onsets, while SNIC/SH offsets show a decrease in spike frequency; and finally, SN/SubH onsets or FLC offsets have constant or random amplitude and ISI. Detailed examples and expected features of each BifM are provided in Figure 2C." Comment 9 Line 52 'comprehensive' is likely too strong. TSD relies on assumptions (eg. 2D systems) Response 9 The sentence "research in this area has mapped out a comprehensive... "has been revised to: "research in this area has mapped out the "taxonomy of seizure dynamotypes" (TSD)..." Comment 10 The reviewers suggest that the the acronym for "Bifurcation Morphology" be modified to something other than "BM" (e.g. BiM, BifM, a different term, or just writing "bifurcation morphology) to avoid conflation with the American english slang use of the acronym BM.

Response 10 We thank the reviewers for their astute observation regarding the acronym "BM." To prevent any unintended associations that might elicit a chuckle, we have changed the acronym to "BifM" throughout the manuscript.
