# Supplementary figures and images for: A New Perspective in Epileptic Seizure Classification: Applying the Taxonomy of Seizure Dynamotypes to Noninvasive EEG and Examining Dynamical Changes across Sleep Stages
Source: eNeuro. 2025 Jan 8;12(1):ENEURO.0157-24.2024. doi: 10.1523/ENEURO.0157-24.2024 (PMC11747977; doi:10.1523/ENEURO.0157-24.2024)

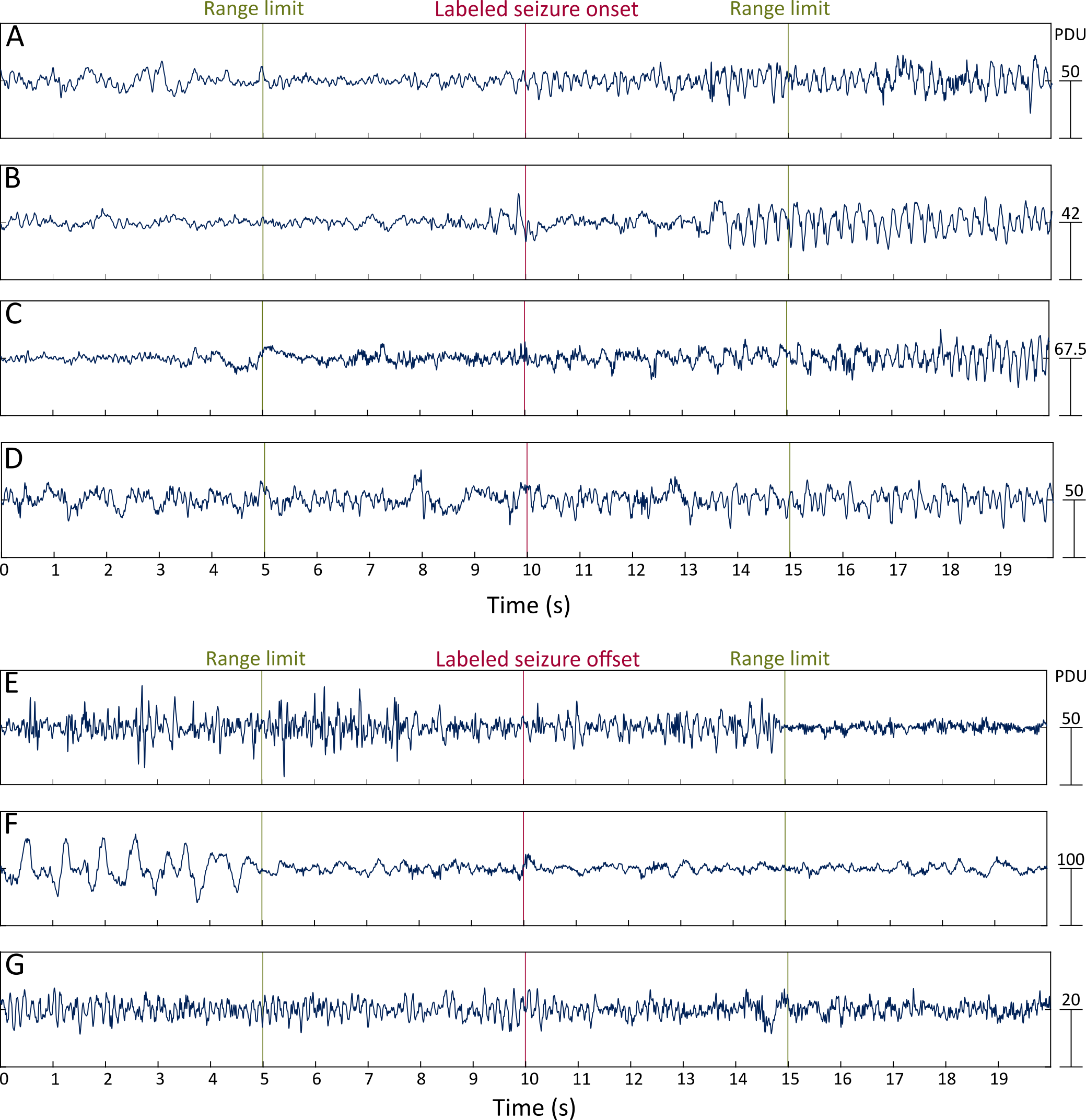

Supplement: Figure 2-1 — Labeling examples. Each component is a linear combination of all electrodes with varying weight ranges; hence, we present the component time series with procedure-defined units (PDUs). Download Figure 2-1, TIF file. [file eneuro-12-ENEURO.0157-24.2024-s002.tif]

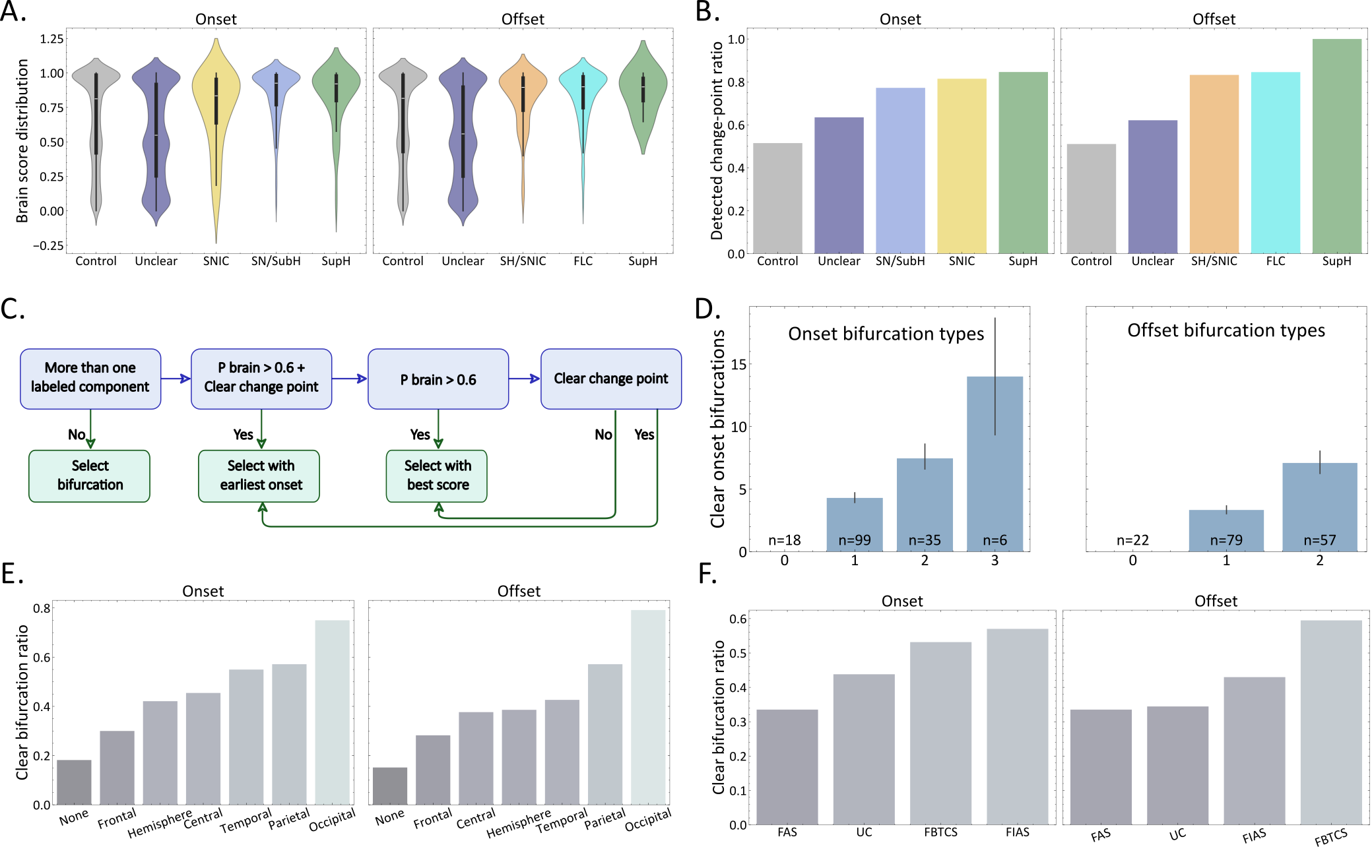

Supplement: Figure 2-2 — Automated measures and detectability characteristics. Download Figure 2-2, TIF file. [file eneuro-12-ENEURO.0157-24.2024-s003.tif]

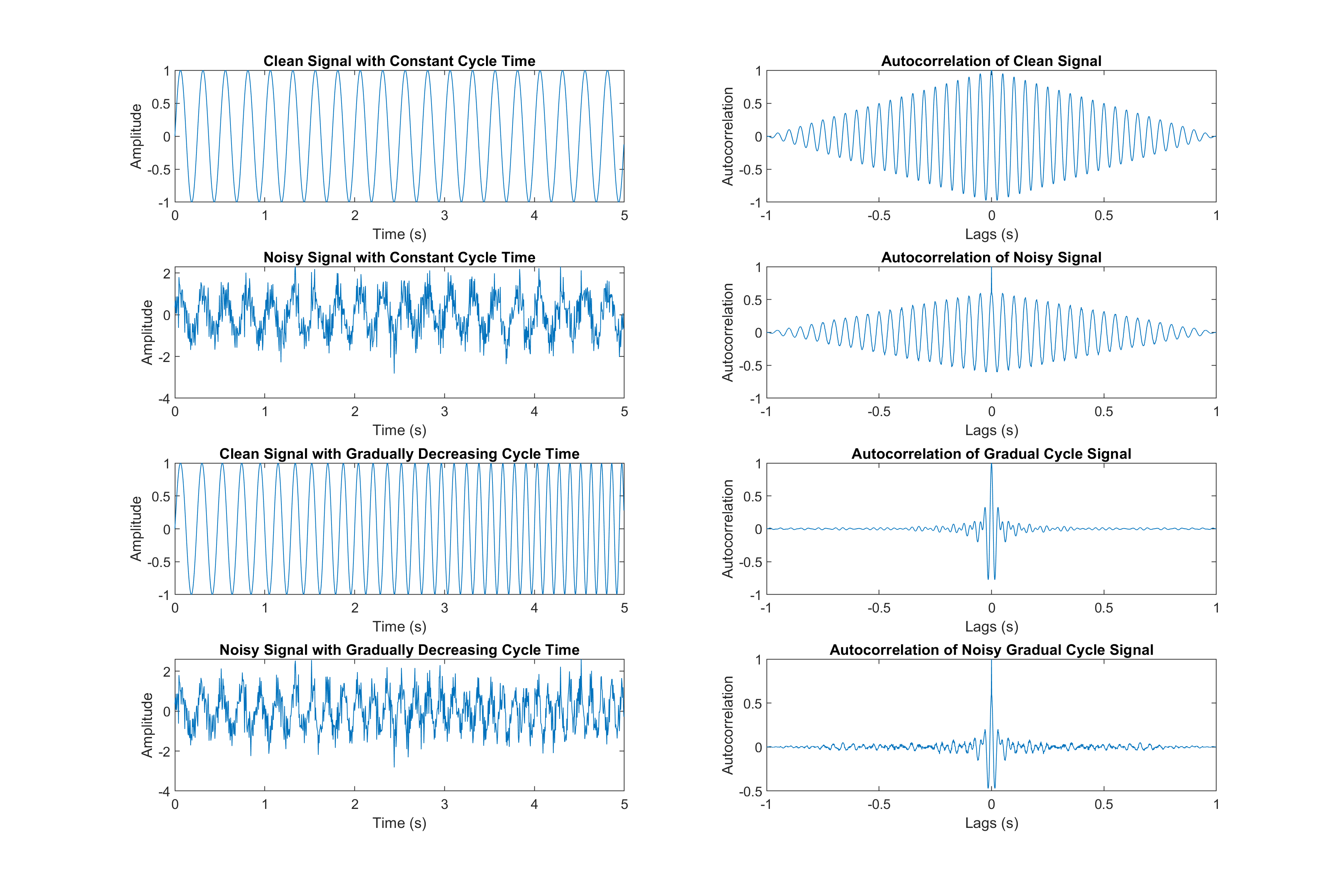

Supplement: Extended Data 1 — This item provides the latest version of the code zipped from our GitHub repository. Within this zipped folder is a README.md file that provides the instructions to run each analysis step presented in this manuscript. Download Extended Data 1, ZIP file. [file eneuro-12-ENEURO.0157-24.2024-s013.zip › miriamguen-surface_eeg_seizure_analysis-main/feature_extraction/signal_simulation_with_autocorrelation.png]

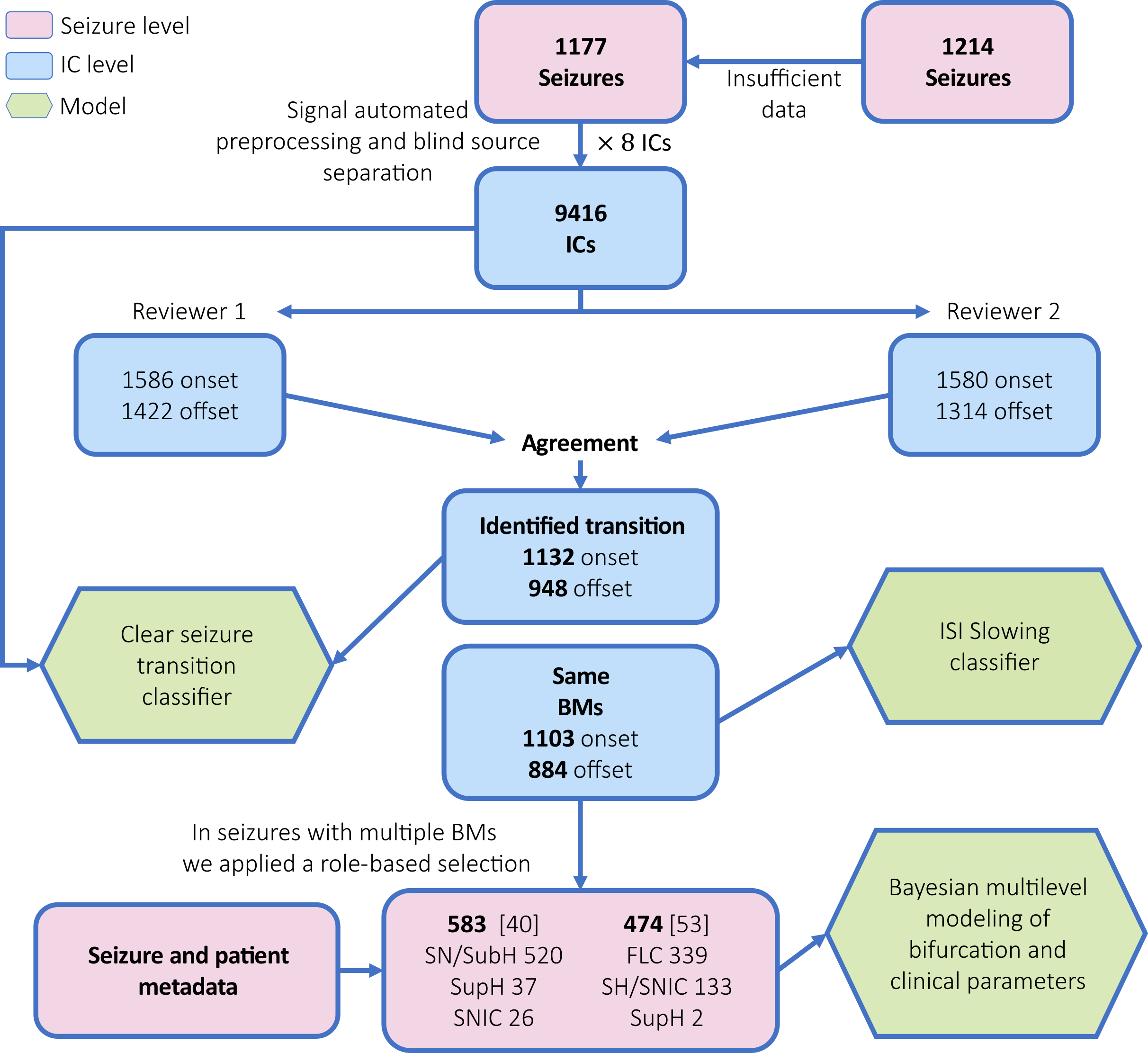

Supplement: Extended Data 1 — This item provides the latest version of the code zipped from our GitHub repository. Within this zipped folder is a README.md file that provides the instructions to run each analysis step presented in this manuscript. Download Extended Data 1, ZIP file. [file eneuro-12-ENEURO.0157-24.2024-s013.zip › miriamguen-surface_eeg_seizure_analysis-main/figures/paper_figures/Figure 1 - The project work flow.png]

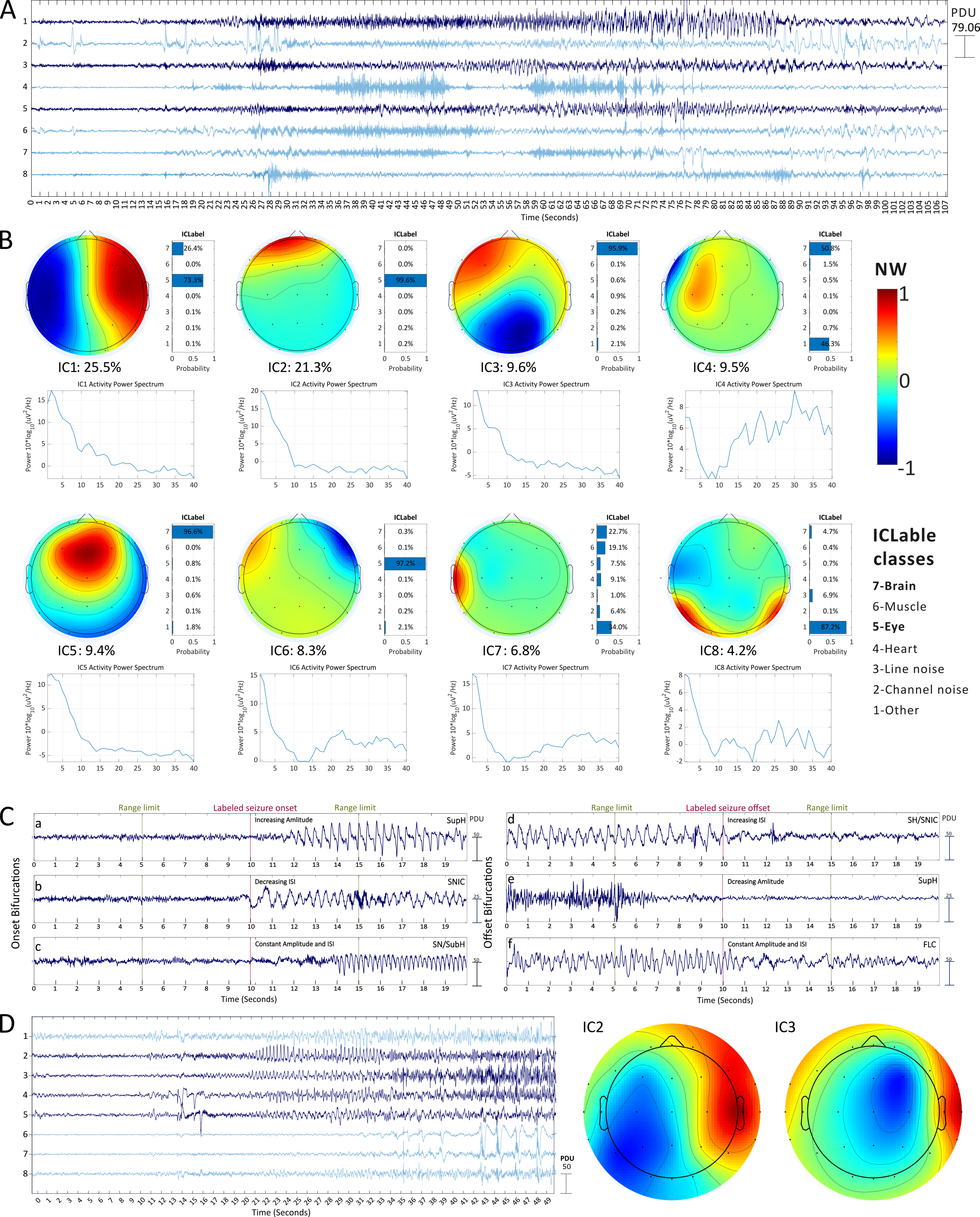

Supplement: Extended Data 1 — This item provides the latest version of the code zipped from our GitHub repository. Within this zipped folder is a README.md file that provides the instructions to run each analysis step presented in this manuscript. Download Extended Data 1, ZIP file. [file eneuro-12-ENEURO.0157-24.2024-s013.zip › miriamguen-surface_eeg_seizure_analysis-main/figures/paper_figures/Figure 2 - Manual labeling interface.png]

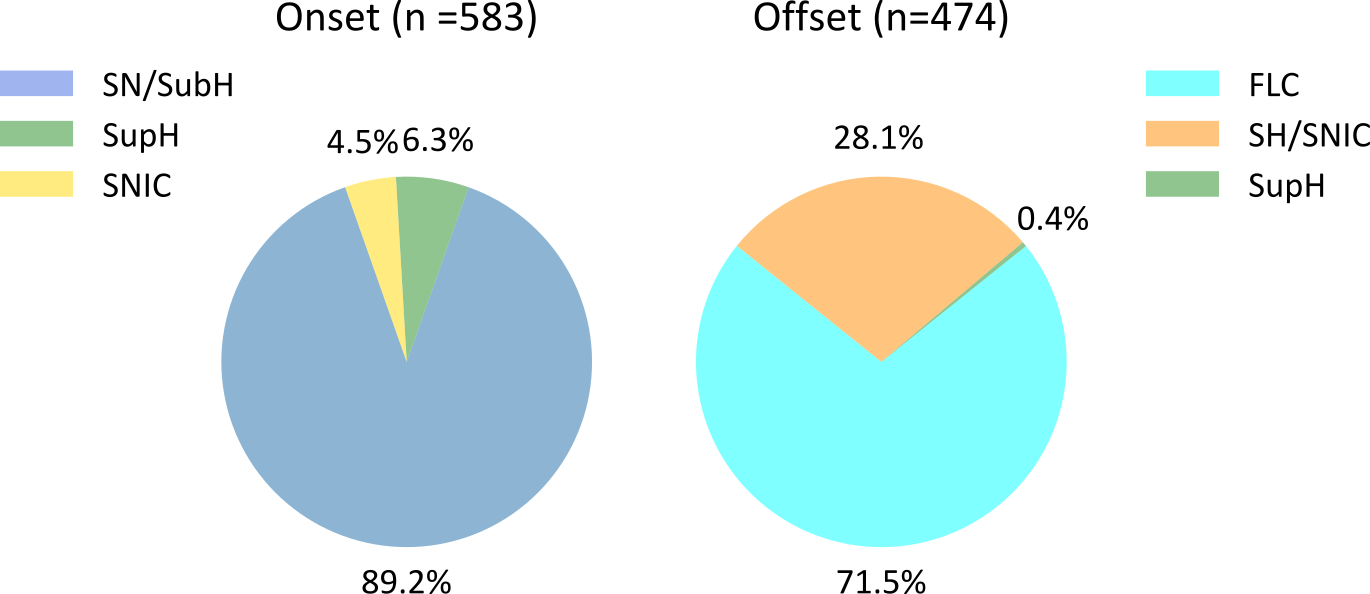

Supplement: Extended Data 1 — This item provides the latest version of the code zipped from our GitHub repository. Within this zipped folder is a README.md file that provides the instructions to run each analysis step presented in this manuscript. Download Extended Data 1, ZIP file. [file eneuro-12-ENEURO.0157-24.2024-s013.zip › miriamguen-surface_eeg_seizure_analysis-main/figures/paper_figures/Figure 3 - label proportions.png]

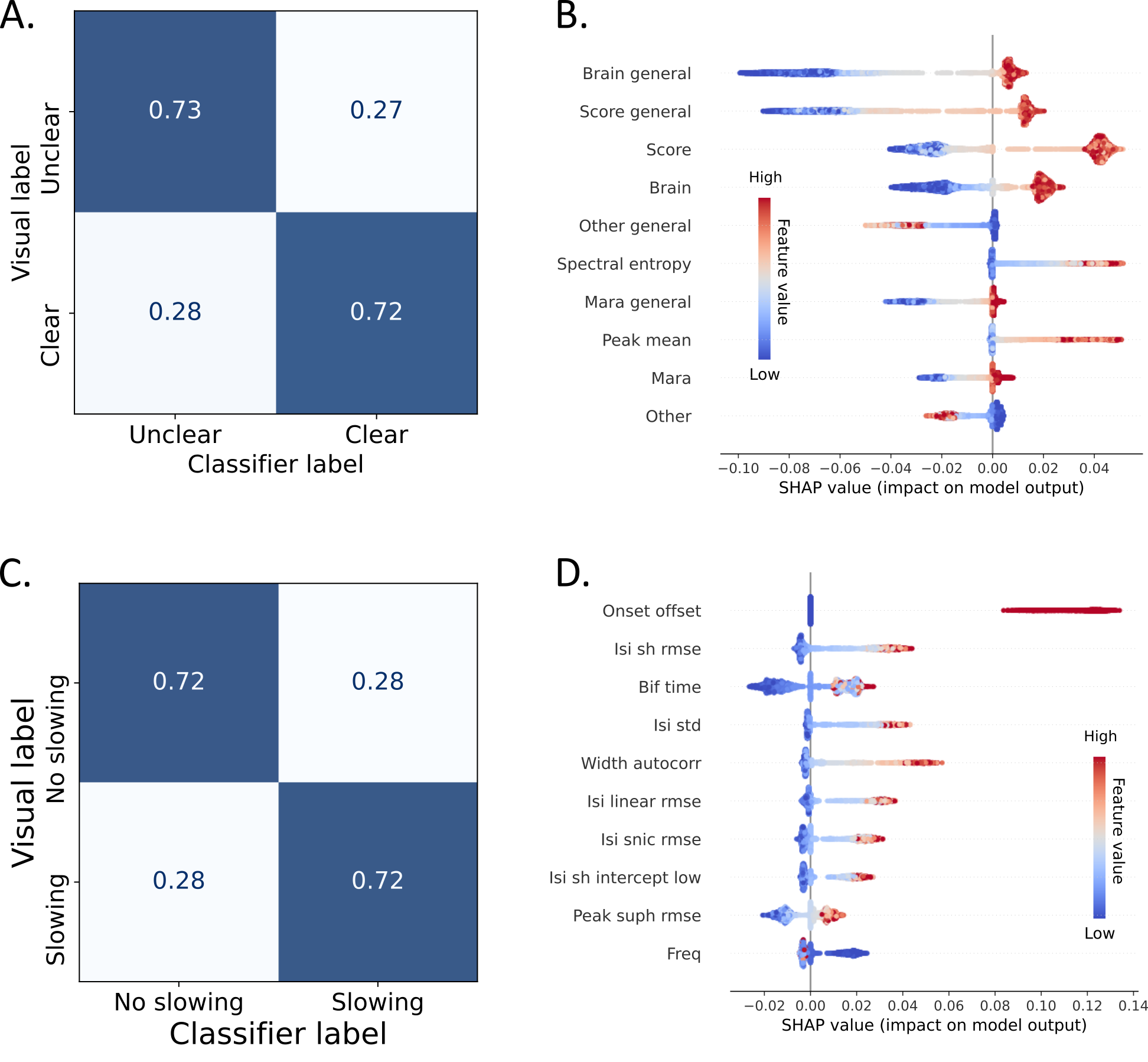

Supplement: Extended Data 1 — This item provides the latest version of the code zipped from our GitHub repository. Within this zipped folder is a README.md file that provides the instructions to run each analysis step presented in this manuscript. Download Extended Data 1, ZIP file. [file eneuro-12-ENEURO.0157-24.2024-s013.zip › miriamguen-surface_eeg_seizure_analysis-main/figures/paper_figures/Figure 4 - classification analysis.png]

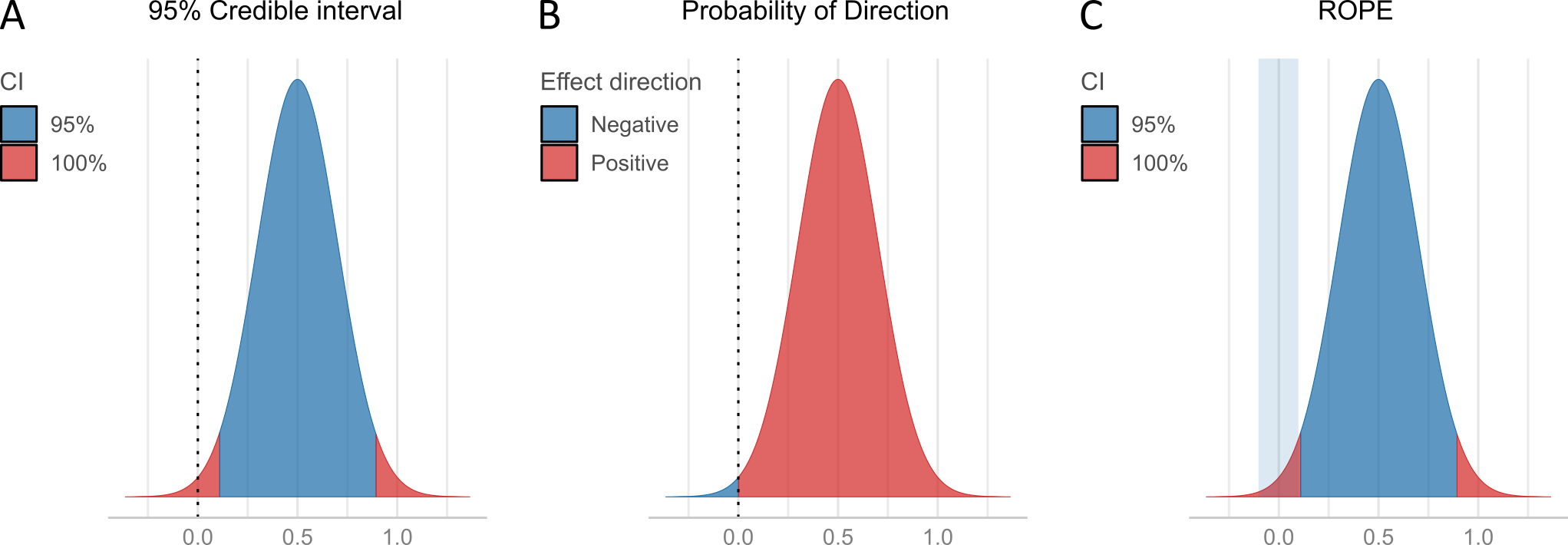

Supplement: Figure 4-1 — The Bayesian measures used for evaluation. This figure provides an intuitive illustration of the measures presented in Table 4. We evaluated the difference between two posterior distributions of bifurcation morphologies, given the tested clinical factors compared to the null hypothesis. The reported measures used in this work are as follows, as suggested by (Makowski et al., 2019). Download Figure 4-1, TIF file. [file eneuro-12-ENEURO.0157-24.2024-s004.tif]
